# Mice Fed Rapamycin Have an Increase in Lifespan Associated with Major Changes in the Liver Transcriptome

**DOI:** 10.1371/journal.pone.0083988

**Published:** 2014-01-07

**Authors:** Wilson C. Fok, Yidong Chen, Alex Bokov, Yiqiang Zhang, Adam B. Salmon, Vivian Diaz, Martin Javors, William H. Wood, Yongqing Zhang, Kevin G. Becker, Viviana I. Pérez, Arlan Richardson

**Affiliations:** 1 Department of Cellular and Structural Biology, The University of Texas Health Science Center at San Antonio, San Antonio, Texas, United States of America; 2 Barshop Institute for Longevity and Aging Studies, The University of Texas Health Science Center at San Antonio, San Antonio, Texas, United States of America; 3 Department of Epidemiology & Biostatistics, The University of Texas Health Science Center at San Antonio, San Antonio, Texas, United States of America; 4 Greehey Children's Cancer Research Institute, The University of Texas Health Science Center at San Antonio, San Antonio, Texas, United States of America; 5 Cancer Therapy and Research Center, The University of Texas Health Science Center at San Antonio, San Antonio, Texas, United States of America; 6 Department of Physiology, The University of Texas Health Science Center at San Antonio, San Antonio, Texas, United States of America; 7 Department of Molecular Medicine, The University of Texas Health Science Center at San Antonio, San Antonio, Texas, United States of America; 8 Department of Psychiatry, The University of Texas Health Science Center at San Antonio, San Antonio, Texas, United States of America; 9 National Institute on Aging, Baltimore, Maryland, United States of America; 10 Linus Pauling Institute, Department of Biochemistry and Biophysics, Oregon State University, Corvallis, Oregon, United States of America; 11 Research Service and Geriatric Research Education and Clinical Center, Audie Murphy VA Hospital (STVHCS), San Antonio, Texas, United States of America; University of Washington, United States of America

## Abstract

Rapamycin was found to increase (11% to 16%) the lifespan of male and female C57BL/6J mice most likely by reducing the increase in the hazard for mortality (i.e., the rate of aging) term in the Gompertz mortality analysis. To identify the pathways that could be responsible for rapamycin's longevity effect, we analyzed the transcriptome of liver from 25-month-old male and female mice fed rapamycin starting at 4 months of age. Few changes (<300 transcripts) were observed in transcriptome of rapamycin-fed males; however, a large number of transcripts (>4,500) changed significantly in females. Using multidimensional scaling and heatmap analyses, the male mice fed rapamycin were found to segregate into two groups: one group that is almost identical to control males (Rapa-1) and a second group (Rapa-2) that shows a change in gene expression (>4,000 transcripts) with more than 60% of the genes shared with female mice fed Rapa. Using ingenuity pathway analysis, 13 pathways were significantly altered in both Rapa-2 males and rapamycin-fed females with mitochondrial function as the most significantly changed pathway. Our findings show that rapamycin has a major effect on the transcriptome and point to several pathways that would likely impact the longevity.

## Introduction

Major advances in the biology of aging have been made over the past two decades due to several novel manipulations that were found to increase the lifespan of invertebrates and rodents. Up until 1996, the only manipulation consistently shown to increase lifespan in rodents was dietary restriction (DR). Because DR was also found to delay/reduce the incidence of most age-related diseases and pathology and to improve most physiological functions, it is generally accepted that DR increases lifespan by delaying aging [1]. In 1996, Brown-Borg et al. [2] reported the first genetic manipulation that increases the lifespan of a mammal; the Ames dwarf mice that have a mutation in *Prop-1*, which resulted in a deficiency in growth hormone leading to growth retardation. Subsequently, a number of genetic manipulations also have been shown to increase the lifespan of mice [3]. In 2009, Harrison et al. [4] reported that feeding mice rapamycin (Rapa) increased the lifespan of mice. This was a major discovery because it demonstrated that a pharmacological manipulation, which potentially could be translated to humans, increased lifespan and appeared to delay aging.

Rapa, which is produced by *Streptomyces hygroscopicus*, inhibits the Target of Rapamycin (TOR) signaling pathway [5]. TOR is a serine/threonine kinase that is a regulatory nexus in response of eukaryotic cells to nutrients, growth factors, and energy status. TOR forms two major complexes in mammals, mTORC1 and mTORC2. The components that make up the complexes are similar, which includes mTOR, mLST8, Deptor, and TTI1/TEL2, with Raptor and PPRAS40 specific for mTORC1 and Rictor, mSIN1, and PPR5/Protor specific for mTORC2 [6]. mTORC1 plays a major role in regulating protein synthesis through the phosphorylation of 4E-BP1 and S6K1. 4E-BP1 controls the cap-dependent translation of mRNA transcripts and S6K1 controls the phosphorylation of riboprotein S6, which is involved in the translation of mRNA transcripts to proteins [7]. In addition to Rapa's well documented effect on cell growth and proliferation [8], decreased mTORC1 signaling is associated with increased autophagy, which could improve protein quality by removing damaged/misfolded proteins. Initially it was shown that Rapa's inhibition was specific to mTORC1 and that mTORC2 was unaffected; however, recent studies suggest that chronic treatment of Rapa may also inhibit mTORC2 [9]. While the functions regulated by mTORC2 are not well known, reports in the literature suggest that mTORC2 has effects on the actin cytoskeleton, plays a role in insulin sensitivity, and regulates AKT signaling [6].

Previous epistasis studies show that inhibition of TOR signaling by genetic manipulation or by feeding Rapa extends the lifespan of invertebrates [10]. The original observation by Harrison et al. [4] showed that Rapa extends lifespan of male and female UM-HET3 mice (generated from four inbred strains) fed Rapa starting at 19 months of age. Subsequently, Anisimov et al. [11] showed that Rapa given intermittently (2 weeks per month) increased the lifespan of female 129/Sv mice, and Miller et al. [12] showed that feeding male and female UM-HET3 mice Rapa starting at 9 months of age increased lifespan. More recently, Zhang et al. [13] reported that C57BL/6 mice fed Rapa starting from 19 months of age also extends lifespan; however, the increase in lifespan in this study was modest compared to that reported by Harrison et al. [4] in UM-HET3 mice. Thus, the effect of Rapa on the lifespan of mice is very robust, suggesting that mTOR signaling plays an important role in aging. Interestingly, DR and Ames dwarf mice, the two manipulations that consistently have been shown to increase lifespan also show a decrease in mTOR signaling [14].

Although Rapa has been shown to alter cell growth/proliferation and autophagy through its inhibition of TOR, we have very little information on the mechanism(s) responsible for Rapa's longevity phenotype. When autophagy genes are blocked in *C. elegans*, lifespan extension by TOR inhibition is prevented and lifespan reverts back to wild type [15]. In *D. melanogaster*, the lifespan extension observed when feeding Rapa is reverted back to control when S6K is overexpressed, 4E-BP1 is inhibited, or the autophagy gene *Atg5* is reduced by RNAi [16]. It has also been shown that knocking out S6K1 (S6k1 is downstream of mTORC1) extends the lifespan of female but not male mice [17]. The goal of this study is to identify pathways/mechanisms by which Rapa extends lifespan in mice by comparing the effect of chronic Rapa treatment on the transcriptome of male and female mice. We show that feeding mice Rapa chronically, beginning at 4 months of age, increased lifespan 16% for females and 11% for males and resulted in major changes in the transcriptome that are associated with 13 pathways that are significantly altered by Rapa in both males and females.

## Materials and Methods

### Animals and feeding regiment

For the chronic Rapa study, male and female C57BL/6 mice were purchased from The Jackson Laboratory (Bar Harbor, ME) and for the 6-months Rapa study, male and female C57BL/6 were purchased from the National Institute of Aging (Baltimore, MD). Two dietary regimens were used in this study: mice fed a commercial chow, LabDiet 5LG6-JL (LabDiet, St. Louis, MO), with Eudragit capsule (control) or 14 ppm encapsulated rapamycin (Rapa) in the food as described by Harrison et al. [4] from 4 months of age for the chronic Rapa study and from 19 months of age for the 6-months Rapa study. Mice in this study were fed the two diets *ad libitum* and maintained on a 12/12 hour light/dark cycle (6:00am/pm lights on/off). For the lifespan study, mice were started on Rapa or control diets at 4 months of age and maintained on these diets until the end of life. Otherwise, mice were maintained on 6-months or chronic dietary regiments until 25 months of age (6 and 21 months of treatment respectively). The mice were sacrificed at the same time of day (9:00 to 11:00am), i.e., the mice were not fasted before collecting the tissues. At which point tissues were collected. Mice were euthanized by carbon dioxide and liver tissues collected, snap frozen in liquid nitrogen, and stored at −80°C until used. At the time of tissue collection, mice were healthy and free of disease as demonstrated by no major loss of weight and normal activity and appearance. All procedures for this study were approved by the Institutional Animal Care and Use Committee at the University of Texas Health Science Center at San Antonio under protocol number: IACUC#10003x.

### Lifespan Study

A total of 175 mice were used in the lifespan study with 40 mice in the control male group and 45 mice in each of the following groups: Rapa males, control females, and Rapa females. Mice were maintained in pathogen-free barrier conditions with 5 mice per cage and were permitted to live out their lives until death due to natural causes without censoring, with the exception of 2 mice, which were terminated early due to fatal neoplastic disease. The mice used in the lifespan study were not disturbed except to check on the mice twice each day and to remove dead mice. Survival analysis were done using Cox proportional hazard and Weibull's accelerated failure time models. The mean, 80%, 50%, 10%, and maximum survival data were calculated for each group from the survival analysis.

### Gompertz mortality analysis

Using the distributions of ages at death, we tested among competing models for acceleration in the rate of increase in mortality based on the Gompertz family of models. The standard Gompertz model is in the form of *u_x_* = *a*e*^b^* ^x^, where *u_x_* is the instantaneous age-specific mortality. Model selection was based on a maximum-likelihood framework implemented in program WinModest [18]. Briefly, four models were considered: the two parameter Gompertz model (the standard Gompertz model, *u_x_* = *a*e*^b^* ^x^), the Gompertz model with a constant additive age-independent term (Gompertz-Makeham model), the three parameter Logistic model (i.e., Gompertz with deceleration), and the Logistic-Makeham with an additive age-independent term. We used the least parameterized model, two-parameter Gompertz, because it provided the best fit for all mortality datasets [19] and the least parameterized model (two-parameter Gompertz) is recommended unless a more parameterized model has a significantly larger likelihood. Significance testing follows a standard method of comparing twice the difference between the likelihoods of the models being compared, which is distributed as chi-square with 1 degree of freedom. Thus, we examined two parameters from the Gompertz model that, together, describe aging dynamics within each treatment group. The first metric was the initial rate of mortality modeled beginning at age 4 months, denoted by the Gompertz parameter *a*. The second metric was the rate of increase in the hazard for mortality, Gompertz parameter *b*, i.e., the mortality rate.

### Rapamycin levels in Blood and Liver tissue

An HPLC system coupled to an AB Sciex API 3200 tandem mass spectrometer with turbo ion spray was used to rapamycin levels in the blood and liver. Rapa and ASCO (internal standard) obtained from LC Laboratories (Woburn, MA) were used in the quantification of Rapa. Approximately 100 ml of blood or 100 mg of liver samples and 0.1 mL of calibrator were mixed by sonification with 10 µL of 0.5 µg/mL ASCO and mixed with a solution containing 0.1% formic acid and 10 mM ammonium formate dissolved in 95% HPLC grade methanol. Supernatants, which were obtained by centrifugation at 15,000 *g* for 5 min at 23°C, were then injected into the LC/MS/MS. The ratio of the peak area of Rapa to that of the internal standard ASCO (response ratio) for each unknown sample was compared against a standard curve using different concentrations of Rapa standard. The concentration of Rapa was expressed as ng/ml for blood and pg/mg of liver tissue protein.

### RNA processing

Mini RNeasy kit (Qiagen, Valencia, CA) was used to extract total RNA from frozen liver (25 mg) following manufacturer's protocols. The RNA quality was assessed by agarose gel and Agilent Bioanalyzer (Agilent Technologies, Santa Clara, CA), and the quantification of RNA was determined using the Nanodrop (Thermo Scientific, Wilmington, DE). Extracted RNA was then processed into cRNA probes for hybridization to arrays using Illumina Total RNA prep kit from Ambion (Life Technologies, Grand Island, NY) following manufacturer's protocols. Liver cRNA probes were then hybridized to Illumina Mouse Ref8 microarrays (V2.0, Illumina, San Diego, CA) following manufacturer's protocol. Hybridized arrays were scanned by the iSCAN system (Illumina, San Diego, CA). Generation and hybridization of probes and scanning of the arrays were done by Dr. Kevin Becker, Unit Head of the National Institute of Aging Gene Expression and Genomics Core.

### Microarray Analysis

Data of the scanned arrays were extracted by Genome Studios software (v 1.6, Illumina, San Diego, CA). The data were then transformed and normalized using log2 transformation and the quantile normalization method in Matlab (2011a, The Mathworks, Natick, MA). For statistical analysis, t-test pairwise comparisons were used. False discovery analysis was then applied to the dataset using the Benjamini-Hochberg (B-H) algorithm as an adjusted p-value. Data processing and statistics were done using Matlab. Analysis of all the detected probes by multidimensional scaling analysis was done using Matlab. For gene analysis, we analyzed microarray data with filtering criteria of adjusted p<0.05 and >15% change in Rapa vs. control in both males and females. Significantly changed genes were then visually analyzed using heatmap analysis in Matlab. Pathway analysis was done using Ingenuity Pathway Analysis (Ingenuity Systems, Redwood City, CA) which uses Fisher's exact test for association of significantly changed genes into pathways and the B-H p<0.05 was then used to determine statistical significance.

### Data Deposition

Microarray data for this study has been deposited in Gene Expression Omnibus with accession number GSE48834 and can be viewed at: http://www.ncbi.nlm.nih.gov/geo/query/acc.cgi?token=nnqvnqugsygaatq&acc=GSE48334.

## Results

**Figure 1:**
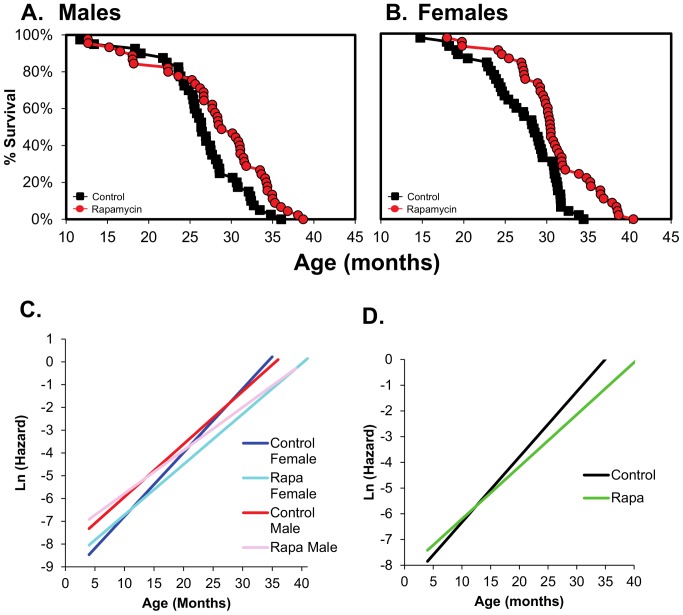
Rapamycin extends the lifespan of male and female C57BL/6 mice fed rapamycin. Survival curves are shown for male (**A**) and female (**B**) C57BL/6 mice fed diets containing eudragit (control, black boxes) or Rapa (red circles) starting at 4 months of age. Forty mice were in the control male group and 45 mice each in the Rapa male, control female, and Rapa female groups. The survival data were statistically analyzed using Cox proportional hazard and Weibull's survival model. The Cox proportional hazard test shows Rapa-fed mice are 47% and 58% less likely to die at the same age as the controls in males (p = 0.004) and females (p<0.001) respectively. The Weibull survival model shows that Rapa-fed male and female have an extension of 11% and 16%, respectively, beyond the controls with p = 0.006 in males and p<0.001 in females. The statistical analysis of the mean, 80%, 50%, 10%, and maximum survival are given in Table S1 in File S1. Ln plots of fitted Gompertz mortality models where the hazard (instantaneous mortality rate) at age x, u_x_ = ae^bx^ with x beginning at 4 months and continuing until the end of life. Models were first fitted to each sex and diet group separately and shown in (**C**). Control males are in red, Rapa males in pink, control females in blue, and Rapa females in cyan. Because the sexes did not differ under control or Rapa treatment using maximum-likelihood estimation, we combined the sexes and compared Rapa to Control diets irrespective of sex (**D**). Combined sex controls in black and combined sex Rapa in green. Pooling of the sexes allowed for identifying the parameter(s) that differ between Rapa and control-fed animals, and indicates that the rate of aging (*b*), but not the initial mortality rate (*a*), differed between Rapa and control mice.

In our lifespan study, male and female mice were given diets containing 14 ppm of rapamycin starting at four months of age. Figures S1 and S2 in File S1 show the body weights and food consumption over the lifespan of the four groups of mice. The mice fed Rapa showed a slightly lower body weight at some ages compared to controls; however, we observed no difference in food consumption. The data in Figure 1A, 1B, and Table S1 in File S1 show that Rapa significantly increased the lifespan of both male and female mice based on the Cox proportion hazard and Weibull's survival analyses. Rapa increased the mean and median survival for females and the 10% survival for both males and females (Table S1 in File S1). The extension of lifespan was slightly greater in females than for the males fed Rapa. For example, when the entire survival curve was analyzed, Rapa was found to increase lifespan 16% in females compared to 11% in males (Figure 1A and 1B). The percent extension in lifespan was also greater in females vs. males for mean (14% vs. 7%) and maximum (19% vs. 8%) survival, and the Rapa-treated females were observed to have a significant increase in survival at earlier ages than males, e.g., 80% survival (Table S1 in File S1). Therefore, Rapa appears to have a greater and earlier effect on lifespan in female than male C57BL/6 mice. This is consistent with the reports using UM-HET3 mice [4], [12]. We fit the distributions of the ages-at-death to the Gompertz family of models using maximum likelihood estimation [18]. From these initial models, the two-parameter Gompertz model provided the most parsimonious fit to the data for all sex and diet groups (Figure 1C and 1D, Table S3 in File S1). Because male and female mice did not differ from each other for the estimated Gompertz parameters when tested within each dietary treatment, we combined the sexes to increase the statistical power of the Rapa versus control comparison. We found that overall, Rapa significantly reduced mortality risk in the Gompertz whole model (p<1×10^−6^) by either reducing the initial mortality rate (at age 4 months), the rate of increasing mortality over the adult life span, or both. We observed no change in initial rate of mortality (p = 0.53); however, the rate of increase in the hazard for mortality, an indication of the rate of aging, approached significance (p = 0.06). Our mortality data is comparable to data reported by Miller et al. [12] for UM-HET3 mice.

**Figure 2:**
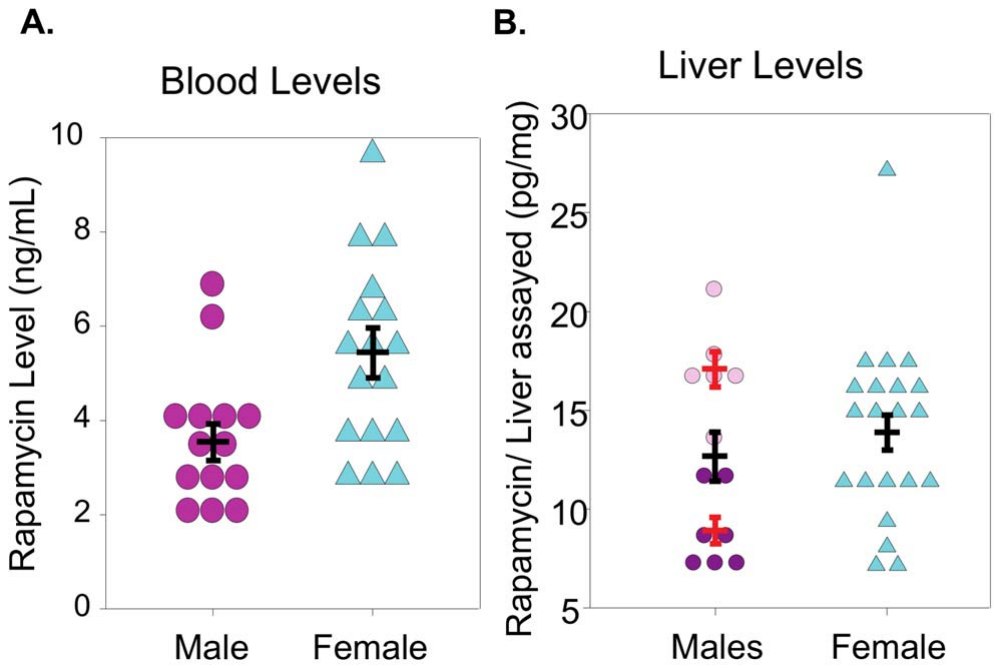
Rapamycin levels in the blood and liver of mice fed rapamycin. Rapamycin levels were measured in mice fed rapamycin starting at 4 months of age using HPLC-mass spectrometry, and the data are presented as ng Rapa per ml of blood or pg Rapa per mg of liver. **Graph A** shows blood levels measured 10 months of age and **Graph B** shows liver levels measured at 25 months of age for liver. The values for each mouse are shown as follows: females (cyan triangles), Rapa-1 males (dark pink circles) Rapa-2 males (pink circles), and total Rapa males in blood (magenta). The red bars indicate mean ±SEM for Rapa-1 and Rapa-2 males while the black bars indicate mean ±SEM for the all the Rapa male and Rapa female mice. The data were statistically analyzed using the student T-tests with blood Rapa male versus Rapa female giving a p-value of p = 0.006, liver Rapa-1 males versus Rapa-2 males giving a p-value of p<0.001, and the liver Rapa male (total) vs. Rapa female giving a p-value of 0.46.

As a first approach into identifying genes and pathways that are altered by Rapa, we compared the liver transcriptome of male and female mice fed the control or Rapa diet, focusing on liver because it is the one of the first tissues to be exposed to Rapa, resulting in the liver being exposed to higher levels of Rapa relative to other tissues. Most important, liver expresses a large and diverse number of metabolic pathways, which allows us to view broadly the effects of Rapa. 25-month-old mice were studied to observe the chronic effect (21 months) of Rapa at a dose that increased lifespan before major changes occurred in survivorship or disease burden, which would complicate the interpretation of our transcriptome data. As shown in Table S2 in File S1, Rapa had no effect on the weight of liver or most tissues in 25-month-old males and females. We found that the levels of blood Rapa measured in 10-month-old males and females were significantly different, while the Rapa levels in the livers of males and females measured at 25-months were not significantly different (Figure 2A and 2B). Interestingly, we observed no difference in mTORC1 signaling in mice (either males or females) fed the control or Rapa diets for 21 months as measured by the ratio of phosphorylation of S6 to total S6 (Figure S3 in File S1), which is in contrast to what we observed when Rapa is given to mice for 6 months [13], [20]. Several other groups have reported that Rapa does not alter mTOR signaling when given chronically over a long period of time [21], [22]. The lack of changes observed in the mTOR signaling by phosphorylated S6 to total S6 ratio could also be due to the lack of synchronization of the mice prior to sacrifice and the Rapa dose studied. We did observe that the TOR transcripts were significantly increased in both Rapa-2 males and Rapa-fed females. Sengupta et al. [23] observed an significant increase in S6 phosphorylation in the liver of mice with age when fasted, but no change when the mice were fed. On the other hand, our microarray data show changes in transcripts for mTORC2 signaling pathway, e.g., *Rictor and Sgk1* are significantly down-regulated, while *Akt* shows no change in expression, and *Pkc1*α expression is increased. Our microarray data suggest that *Pkc1α* gene expression is up-regulated (in Rapa-2 male). These data are in agreement with Habib et al. [24], who found that sirolimus (rapmaycin) activates PKC1α in endothelial cells. Based on our transcriptome data, it appears that the mTORC2 signaling pathway may be reduced because of the down-regulate of Rictor, which is needed in the formation of mTORC2, which agreed with previous data from Lamming et al. [9].

**Figure 3:**
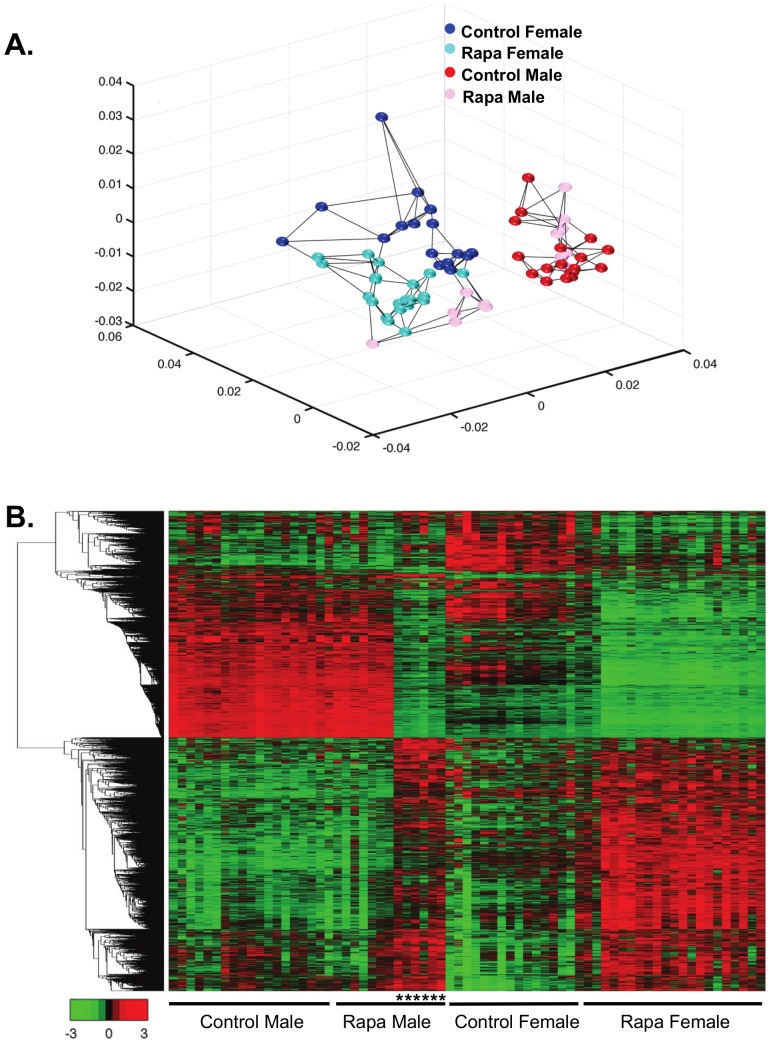
Multidimensional scaling and heatmap analyses shows the separations of Rapa and control groups. Multidimensional scaling analysis was conducted using all the probes detected (25,697 probes) on the microarrays (**A**). Control males are shown in red, Rapa males in pink, control females in blue, and Rapa females in cyan. Each dot represents one sample and the lines indicate the three nearest neighbors. In the females, the Rapa mice segregate separately from the control mice, while in the males, 7 Rapa mice group similar to the control males and 6 of the Rapa males do not group with the control males. From this analysis, we separated the Rapa males into two groups for subsequent analysis, Rapa-1 males as the group that appears similar to the control males and Rapa-2 males as the group that appears different from the control males. The heatmap analysis shows the expression of all transcripts that were observed to change significantly with Rapa in both male and female mice (**B**). The genes were clustered using linkage hierarchical cluster with Euclidean distance. The transcript expressions are shown with red for high, black for middle, and green for low expression. The male mice in Rapa-2 are shown by the asterisks. A list of the transcripts that significantly changed with chronic Rapa feeding is shown in the same top to bottom clustering order as the heatmap in File S2, tab1.

We first used the multidimensional scaling analysis (MDS, Figure 3A) to compare the transcriptomes of male and female mice fed the two diets. MDS is an unbiased analysis that allows us to determine which of the four groups are similar and different. Using all the probes detected (25,697), we clearly observe gender differences in the transcriptome; the control-fed male and female mice form separate groups. In the females, the Rapa-fed mice appear as a group that is distinctly separate from the control-fed mice. In contrast, the Rapa males appear in two groups; 7 mice appear similar to the control males and 6 mice similar to the Rapa females. We observed no difference in body weight, body composition, and mTOR signaling between Rapa-1 males (the group similar to control males) and Rapa-2 males (the group that differs from control males). However, we did observe a difference in Rapa levels. Rapa-2 males had a significantly higher level of Rapa in the liver than Rapa-1 males (Figure 2B). Interesting, neither the mean level of Rapa nor the variation in Rapa levels was different for male and female mice fed Rapa at 25 months of age.

Genes whose expression were significantly changed by feeding Rapa were identified using a false discovery rate of adjusted p<0.05 and >15% change. In Rapa females, 4761 transcripts changed significantly from control females; 2504 up-regulated and 2257 down-regulated. For the Rapa males, 288 transcripts significantly changed from control males with 159 up-regulated and 129 down-regulated. Because of our MDS data, we compared the control males to the Rapa-1 and Rapa-2 male groups. Rapa-1 males showed only 28 genes significantly changed from control males (9 up-regulated and 19 down-regulated, Figure S4A and S4B in File S1). When we compared Rapa-2 males to control males, 4042 transcripts were significantly changed with 2092 up-regulated and 1950 down-regulated (Figure S4C and S4D in File S1). Over 60% of the transcripts (2498) that changed in Rapa-2 males were also significantly altered in Rapa females: 1178 (56%) of the transcripts that increased and 1320 (68%) that decreased in Rapa-2 males.

**Figure 4:**
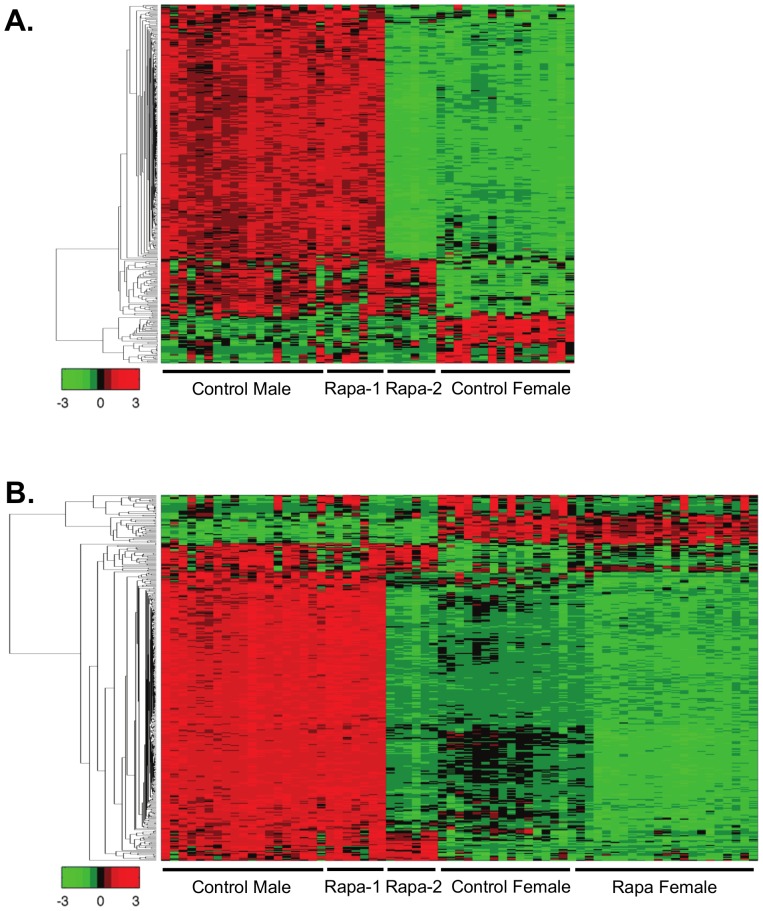
Heatmap analysis of the transcriptome for gender differences. We identified the 372 transcripts (333 higher in males and 39 higher in females that were significantly different in control male and female 25-month-old mice using a filtering criteria of adjusted p-value <0.01 and log2 change greater than 1 or −1. The expression of these transcripts are shown in a heatmap analysis format for Control male, Rapa-1 male, Rapa-2 male, and Control female mice (**A**). The differences in the gender specific transcripts highlight that Rapa-2 male mice are different from control and Rapa-1 male and that the changes appear similar to the females. However when Rapa female mice is added to the heatmap analysis, it appears that the gender specific transcripts in Rapa-2 male are actually more similar to the Rapa female mice (**B**). The genes were clustered using linkage hierarchical cluster with Euclidean distance, and the expression of each of the transcripts are shown with red for high, black for middle, and green for low expression.

Heatmap analysis of the transcriptome data allowed us to visually compare the levels of transcripts in each of the mice in the four groups. Figure 3B shows that the patterns of change in Rapa females are different from control females. As would be predicted from the MDS analysis, the pattern of gene changes in the Rapa-2 males differed from the Rapa-1 males and became similar to the Rapa female while the patterns of intensities in Rapa-1 males are almost identical to control males. When we compared the genes that are gender specific (see heatmap in Figure 4), we observed that the expression of the gender specific transcripts in Rapa-1 males was identical to the control males; however, the pattern of expression of these transcripts for the Rapa-2 males was strikingly similar to the control females and Rapa females, suggesting that the gender specific differences in the liver transcriptome were reduced in the Rapa-2 males. Previous reports show that Rapa treatment in humans reduced testosterone levels [25] and Rapa-fed mice show testicular degeneration [26].

**Figure 5:**
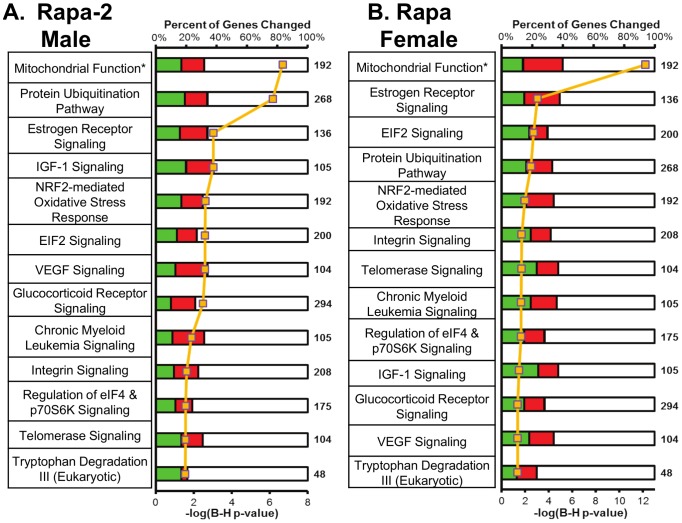
Pathway analysis indicates that male and female mice fed Rapa share 13 pathways. Pathway analysis was conducted using IPA for significantly changed transcripts in Rapa-2 males and Rapa females relative to respective sex controls with adjusted p<0.05 and 15% change. IPA used Fisher's exact test to place genes into pathways, and we also used a FDR cutoff of B-H p<0.05 to show the highly significant pathways. The 13 shared pathways ranked by B-H p-value are shown for Rapa-2 males (**A**) and Rapa females (**B**). For the graphical portion of A and B, the yellow line indicates the –log(B-H p-value). Red indicates up-regulated and green represents down-regulated genes significantly changed in Rapa compared to control, and white indicates the percentage of genes not significantly changed in the pathway relative to the total number of genes in that pathway (bolded number). Twenty-four other pathways were observed to change significantly in Rapa-2 males: acute phase response signaling, hypoxia signaling, PI3K/AKT signaling, remodeling of epithelial adherens junctions, sertoli cell-sertoli cell junction signaling, renal cell carcinoma signaling, JAK/Stat signaling, IL-3 signaling, myc mediated apoptosis Signaling, germ cell-sertoli cell junction signaling, pancreatic adenocarcinoma signaling, insulin receptor signaling, non-small cell lung cancer signaling, Rac signaling, small cell lung cancer signaling, PTEN signaling, prostate cancer signaling, neurotrophin/TRK signaling, PAK signaling, RAR activation, NGF signaling, clathrin-mediated endocytosis signaling, huntington's disease signaling, and TCA cycle II. Six other pathways were observed to change significantly in Rapa females: systemic lupus erythematosus signaling, mTOR signaling, actin nucleation by ARP-WASP complex, superpathway of methionine degradation, glutathione-mediated detoxification, and oncostatin M signaling. A list of the pathways that significantly changed with chronic Rapa feeding is shown in in File S2, tab2 for Rapa-2 male; tab3 for Rapa female and the transcripts that change for the 13 shared pathways are found in File S3. The asterisk indicates that the mitochondrial function pathway is indicated as mitochondrial dysfunction in IPA. The mitochondrial dysfunction pathway described in IPA is somewhat misleading because it contains genes involved in mitochondrial function in general, e.g., genes in the electron transport chain complexes and redox and antioxidant enzymes as well as other genes related to mitochondrial function.

To identify the pathways that were changed with Rapa, we used ingenuity pathway analysis (IPA), which places genes into pathways determined from the literature. We observed that 37 pathways significantly changed in Rapa-2 males and 19 pathways significantly changed in Rapa females and these are listed in File S2 tab2 and tab3. In Figure 5, we show the 13 pathways shared by Rapa-2 males and Rapa females, ranked by the lowest B-H p-value. We validated the changes in genes in the mitochondria function and estrogen signaling pathways using quantitative real-time PCR. In the mitochondrial function pathway, we show that transcripts for *Glrx2* and *Cox6A2* were down-regulated while *Cyb5r3a* and *Cyc1* were up-regulated in Rapa-2 males and Rapa females (Figure S5A in File S1). In the estrogen receptor signaling pathway, *Esr1*, *Sra1*, and *Kat2b* were all down-regulated in Rapa-2 males and Rapa females similar to what was observed in the transcriptome analysis (Figure S5B in File S1). The expression of *Stat3* and Pck1 (Figure S5C in File S1), which are genes downstream of the estrogen receptor signaling, are also down-regulated in both Rapa-2 males and Rapa females.

We also used Database for Annotation, Visualization and Integrated Discovery (DAVID) bioinformatics resources [27], [28] to identify the biological function, relevance, and significance of the transcripts that were significantly altered by Rapa. DAVID is a collection of analysis tools that enhance the identification of the biological relevance of transcripts by gene enrichment type analyses to highlight transcripts that are highly represented. Transcripts were first sorted into several list functions such as gene ontology categories, keywords, sequence function, or pathway databases such as KEGG. Transcripts in those lists were then enriched and the relevant terms in those lists are shown with an enrichment score. The higher the enrichment score, the more significance those transcripts have in the representation of the changes observed. Using DAVID, transcripts in the mitochondria, lumen/membranes, and protein degradation were observed to be significantly enriched in Rapa-2 males and Rapa females as shown in Table S4 in File S1 and File S4. These data are consistent with the pathways identified by IPA analysis.

**Figure 6:**
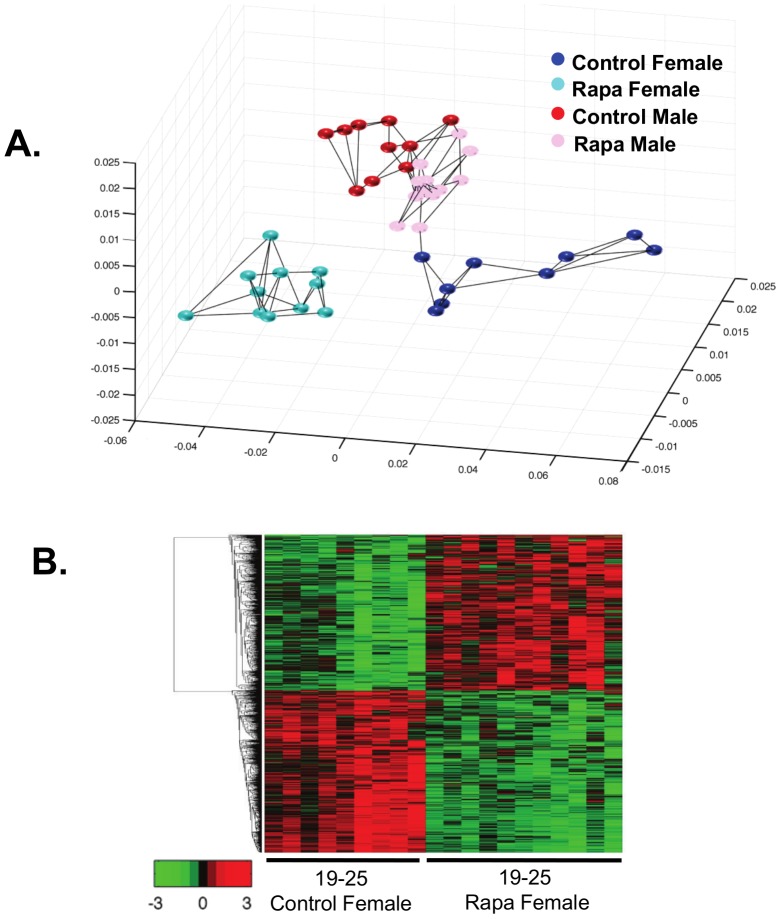
Multidimensional scaling and heatmap analysis show that 6-months Rapa females are separate and different from control females. Multidimensional scaling analysis of the transcriptome data from the 42 mice fed Rapa 6-months from 19 to 25 months of age was conducted using all the probes detected (17,082 probes) on the microarrays (**A**). Control males are shown in red, Rapa males in pink, control females in blue, and Rapa females in cyan. Each dot represents one sample and the lines indicate the three nearest neighbors. Heatmap analysis of all the transcripts that are significantly altered by 6-months Rapa female compared to control females are shown (**B**). The genes were clustered using linkage hierarchical cluster with Euclidean distance and the expression of transcripts are shown with red for high, black for middle, and green for low expression. A list of the transcripts that significantly changed with 6-months Rapa feeding is shown in the same top to bottom clustering order as the heatmap in File S2, tab4.

Because our data show that feeding male and female mice Rapa for 21 months resulted in major changes in the transcriptome, we also studied the effect 6-months Rapa on the transcriptome. Male and female mice at 19 months of age were fed 14 ppm of Rapa for 6 months, and the transcriptome of the livers of these mice analyzed at 25 months of age. Zhang et al. have characterized these mice [13]. Only 100 transcripts were found to change significantly in the 6-months Rapa males compared to control males: 32 increased and 68 decreased (Figure S6A and S6B in File S1). In 6-months Rapa females, 1427 transcripts changed significantly compared to control females: 675 increased and 752 decreased. Only 7 genes (1%) that changed in Rapa males were similarly decreased in the Rapa females. Figure 6A shows the MDS analysis of the genes significantly altered by feeding Rapa for 6 months in male and female mice. The control male and female mice form separate groups, and the Rapa females segregate as a separate group from the control females. However, the male mice fed Rapa grouped together with the male mice fed the control diet. Figure 6B shows the heatmap of genes that significantly changed in the female mice fed rapamycin for 6 months compared to the control females. The difference in the pattern of gene expression between the female mice fed Rapa and those fed the control diet is striking, agreeing with the MDS analysis.

**Figure 7:**
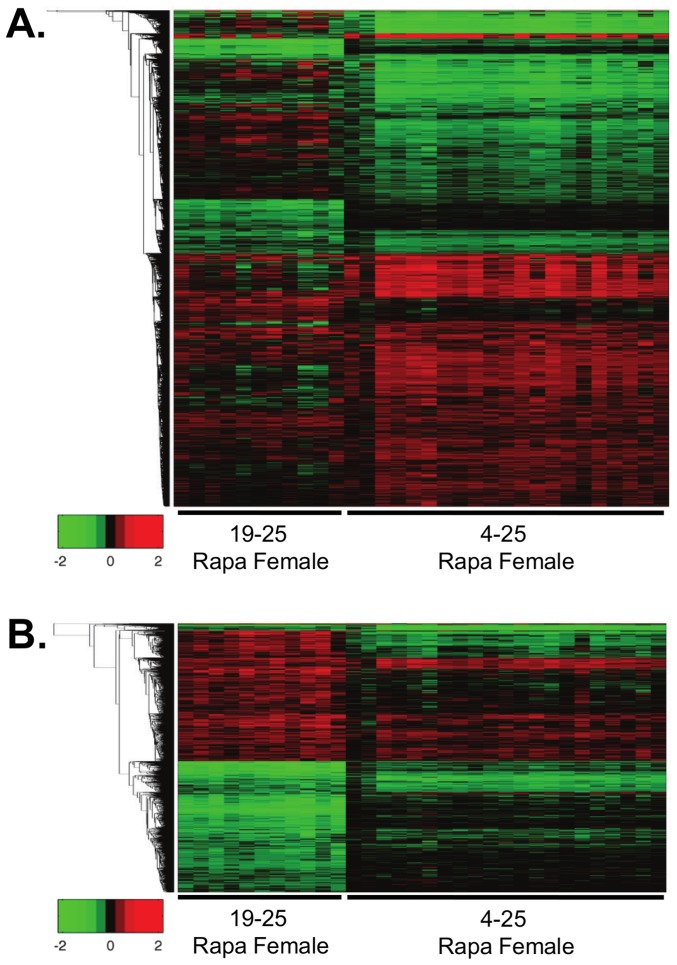
Relative heatmap analysis indicates high degree of similarity between 6-months and chronic Rapa feeding in female mice. Heatmap analysis of individual, 25-month-old female mice fed Rapa for 6 (6-months) or 21 (chronic) months compared to the respective average of the control females are shown. The transcripts that are significantly changed in 6-months or chronic Rapa females are shown in (**A**) and the transcripts that only significantly change in 6-months Rapa females are shown in (**B**) compared to chronic Rapa females. For this heatmap analysis, red indicates up-regulated transcripts, green indicates down-regulated transcripts, and black indicates little or no change. The transcripts were plotted using log2 values and the heatmap scales from a change of −2 to 2 log2 change. A list comparing the transcripts that significantly changed with 6-months or chronic Rapa feeding is shown in the same top to bottom clustering order as the heatmap in File S2, tab6.

We next compared the effect of 6 and 21 months of Rapa treatment on the liver transcriptome. In these analyses, we used the female mice because Rapa changed the levels of only a few transcripts in male mice. After matching the microarray datasets for detected transcripts with 6 months and 21 months of Rapa treatment in females, we observed 1427 transcripts changed in the 6-months Rapa female group, 675 increased and 752 decreased; 4761 transcripts changed with chronic Rapa female group, 2504 increased and 2257 decreased. We observed that 577 transcripts changed in similar directions in both the 6-months and chronic Rapa treated females, i.e., 40% of the transcripts that changed in the 6-months Rapa female mice. The heatmap analysis of gene expression in Figure 7 for the two groups of female mice shows similar, but not identical, patterns of expression. We observed that 6 months of Rapa treatment significantly altered 3 pathways in the females: mitochondrial function and protein ubiquitination, which were shared with the female and Rapa-2 mice fed Rapa for 21 months, and RAN signaling (Figure S6C in File S1).

## Discussion

Harrison et al. [4] were the first to show that rapamycin increased lifespan of UM-HET-3 mice when Rapa treatment was initiated at 19 months of age, e.g., a 9% and 14% increase in the lifespan of male and female mice, respectively. In a follow-up study, Miller et al. [12] showed a similar extension of lifespan in male and female UM-HET3 mice when Rapa feeding was initiated at 9 months of age. Interestingly, Zhang et al. [13] observed only a modest increase in lifespan of C57BL/6 mice when Rapa feeding was begun at 19 months of age. The lifespan of female mice increased only 6%, and Rapa had no significant effect on the lifespan of male mice. In this study, we show that Rapa treatment initiated at 4 months of age increased the lifespan of both male and female C57BL/6 mice to a level similar to that observed in UM-HET3 mice; lifespan was increased 11% for male and 16% for female C57BL/6 mice. Gompertz mortality analysis of our data suggest that the significantly decreased mortality hazards in Rapa-fed mice is due to a reduction in the rate of aging in the C57BL/6 mice, which is comparable to what Miller et al. [12] observed in UM-HET3 mice. In the studies with UM-HET3 and C57BL/6 mice, the Rapa dose (14 ppm) and diet composition were the same. Thus, C57BL/6 mice appear to require longer period of exposure to 14 ppm Rapa to show an increase in lifespan similar to that observed in UM-HET3 mice. We believe this difference in response to rapamycin most likely arises because levels of Rapa are much lower in the C57BL/6 compared to UM-HET3 mice. The blood levels of C57BL/6 mice fed 14 ppm Rapa in our study were 3–5 ng/ml compared to 13.4±2.6 [26] or 60–70 ng/ml [4] for UM-HET3 mice. Because drug metabolism and clearance has been shown to be high in C57BL/6 mice compared to other inbred lines of mice [29], we speculate that the lower levels of Rapa in the C57BL/6 mice are due to increased metabolism/elimination of Rapa. The goal of our study was to identify potential pathways that might be responsible for Rapa's life-extending action by probing its effect on the transcriptome. We focused on the liver because it is one of the first tissues exposed to Rapa, and it is relatively homogeneous; hepatocytes constitute approximately 80% of the liver volume and thus potential differing effects of Rapa on different cell types are minimized. Most important, liver expresses a large and diverse array of metabolic pathways, which allows us to evaluate the effects of Rapa on the expression of a broad range of transcripts.

Our data show 21 months of feeding Rapa has a major effect on the transcriptome of female and male (Rapa-2 group) mice. Over 6,300 transcripts were altered in female and Rapa-2 male mice with ∼40% of these transcripts shared. From our transcriptome data, we identified 13 pathways that were shared between the females and Rapa-2 males fed Rapa for 21 months and two (mitochondrial function and protein ubiquitination) of these 13 pathways were also altered in female mice fed Rapa for 6 months. Three of the 13 pathways are potentially involved in the anti-cancer action of Rapa, e.g., estrogen receptor signaling, VEGF signaling, and chronic myeloid leukemia signaling. Our data suggest that Rapa down-regulates the estrogen receptor signaling pathway, e.g., transcripts for *Esr1* and the co-activator *Sra1* were significantly reduced, while the transcript for *Spen*, an inhibitor of *Sra1*, were significantly increased in female and Rapa-2 males chronically fed Rapa. Estrogen receptor alpha transcript levels have been reported to be reduced by Rapa in C2C12 myoblasts [30], and estrogen receptor positive breast cancer lines show reduced growth when treated with Rapa [31]. VEGF signaling, which controls vascular function and angiogenesis that is necessary for cancer progression, appears to be decreased in Rapa-fed male and female mice, e.g., the transcripts for *Vegfa* and *Flt1*, the VEGF receptor-1, are decreased. Guba et al. [32] showed that Rapa inhibits adenocarcinoma tumor growth and migration by decreasing VEGF production and signaling. In chronic myeloid leukemia signaling, the expression of *p53* and *Smad3* are increased in both males and females fed Rapa. *p53* is a well-known tumor suppressor that controls the G1/S checkpoint in cell cycle and induces DNA damage repair. *Smad3* is a downstream regulatory element of the Tgf-β pathway that has been shown to inhibit cell growth [33].

Interestingly, six of the 13 pathways have been shown to modulate aging, e.g., mitochondrial function, protein ubiquitination, NRF2-mediated oxidative stress response pathway, glucocorticoid receptor signaling, IGF-1 signaling, eIF2 signaling, and the regulation of eIF4 & p70S6K signaling. Mitochondrial function pathway was the pathway most significantly changed by Rapa in the female and Rapa-2 mice, it is the only pathway significant at B-H p<1×10^−6^. The majority of the transcripts that change in complex I and II transcripts decrease while complex III, IV, and V transcripts that change increase in chronic Rapa-fed female and Rapa-2 male mice. We also observed a decrease in the expression of genes involved in redox regulation, e.g., a significant decrease in transcripts for *Sod2*, *Glrx2*, and *Prdx5* were observed in both female and Rapa-2 mice fed Rapa chronically. In 6-months Rapa females, the genes in complex I, II, IV, and V are down-regulated, while complex III is not changed. *Clpp*, an ATP-dependent peptidase in the inner mitochondrial membrane, which is involved in the activation of the mitochondrial protein unfolding response [34], is increased in mice fed Rapa. In 6-months Rapa females, genes in complex I, II, IV, and V were decreased along with *Glrx2* and *Prdx5*. Currently, there is little information about the effect of Rapa on mitochondria function in animals. Studies with cells in culture indicate that Rapa treatment alters mitochondrial function, e.g., reduced oxygen consumption [30], [35] and decrease mitochondrial membrane potential and ATP synthesis [35].

Protein ubiquitination, which signals specific proteins for degradation through the proteasome, appears to be decreased in chronically Rapa-fed mice, e.g. several ubiquitin-conjugating E2 transcripts such as *Ube2a, Ube2d3, Ube2g1, Ube2q1* and ubiquitin-ligase E3A are decreased. Proteasome subunits such as *Psmc1, Psmd1, Psmd10, Psmd14, Psme7, and Psme9* are also decreased in Rapa-2 males and females. In 6-months Rapa females, ubiquitin-conjugating E2 transcript *Ube2j1* and proteasome subunits *Psmb10, Psmb5, Psmb7, Psmc6, Psmd6, Psmc7, Psmd11, Psmd12*, and *Psmd14* are decreased suggesting a decrease in protein ubiquitination as well. Zhang et al. [13] reported that proteasome activity was reduced in liver as well as other tissues in 19-month old female mice fed Rapa for 6 months. They also observed that the decrease in proteasome activity was paralleled by increased autophagy, suggesting Rapa treatment shifts protein degradation from the proteasome pathway to the autophagy pathway. It has been shown previously that protein degradation through the proteasome and autophagy pathways is coordinated in a way that changes in one pathway are countered by changes in the opposite direction in the other pathway, e.g., the chronic inhibition of proteasome are paralleled by increased autophagy [36].

The up-regulation of the NRF2 pathway is believed to be in extending lifespan and delaying aging. For example, NRF2 has been suggested to partially be responsible for the increased resistance to oxidative stress observed in DR mice [37]. In addition, overexpression of *skn-1*, the *Nrf1/2/3* homolog in *C. elegans*, extends lifespan and increase stress resistance [38]. Changes in the levels of several transcripts in the NRF2 signaling pathway suggest that this pathway is reduced in chronically fed Rapa-2 males and females. First, *Nrf2* transcripts are decreased, and the transcript level of *Keap1*, the inhibitor of Nrf2, are increased. Second, the anti-oxidative responsive genes regulated by NRF2 are down-regulated (*Gst, Sod2*, *Cat*, *Fth1*, and *Prdx1* transcript levels are significantly reduced). Also, the levels of the transcript for *Gclm*, which is the rate limiting enzyme in glutathione biosynthesis, is reduced in the mice fed Rapa. A reduction in NRF2 signaling would be predicted to result in tissues/cells becoming more sensitive to a variety of environmental stresses. Our preliminary data show that primarily cultures of mouse fibroblasts pre-treated with Rapa are more sensitive to a variety of stresses, e.g., hypochlorous acid, paraquat, and *t*-butyl hydroperoxide (Figure S7 in File S1).

The identification that the IGF-1 signaling pathway as one of the pathways significantly altered by Rapa is interesting because research over the past decade points to Igf-1 being important in aging, e.g., circulating IGF-1 levels are reduced by DR, mutations in *daf2*, the homolog of *Igf-1* in *C. elegans*, increase lifespan [39] and female mice deficient in the IGF-1 receptor show increased lifespan [40]. *Igf-1* transcript is increased in both Rapa-2 males and Rapa females, suggesting that Rapa treatment induces IGF-1 synthesis by the liver, which is the tissue responsible for the production of circulating levels of IGF-1. Igf binding protein-1, which binds IGF-1 maintaining it in an inactive state [41], transcripts are decreased significantly in Rapa-2 males and decreased in Rapa females but not significantly. Igf binding protein 7, which also binds with a higher affinity to insulin, transcripts are decreased in both Rapa-2 males and Rapa females. Thus, our data suggest that circulating levels of IGF-1 accessible to other tissues is higher in the mice fed Rapa. While an increase in IGF-1 signaling may not be comparable to what has been observed with DR and mutants in Igf-1/Igf-1-receptor, increased IGF-1 signaling could have a positive effect on the aging brain because IGF-1 has been shown to improve brain synaptic structure, plasticity, and functions [42].

Two of the six pathways potentially involved in aging are related to protein synthesis: eIF2 signaling and the regulation of eIF4 & p70S6K signaling. The eIF2 and eIF4 & p70S6K signaling pathways overlap and play a major role in the regulation of translation. One would expect the eIF2 and eIF4 & p70S6K signaling pathways to be reduced in liver tissue of our mice treated with Rapa because mTOR, which regulates translation, is inhibited by Rapa. We observe a significant reduction in the transcript levels of a number of ribosomal proteins in the 60S (*Rpl4, Rpl23, Rpl39, Rpl27a, and Rpl7*) and 40S (*Rps3* and *Rps3a*) ribosome subunits in Rapa-2 males and Rapa females. A similar effect was also observed in 6-months Rapa treated females, which is consistent with previous reports that reduced mTOR signaling decreases ribosome biogenesis [43]. We also observed changes in the levels of transcripts for proteins that regulate the initiation of protein synthesis. For example, the transcript levels of *Eif3*, which activates initiation by facilitating the initial binding of the 40S ribosome subunit to mRNA, was significantly reduced; while the transcript for *Eif2β*, which facilitates the binding of tRNA and the 60S ribosome to the 40S initiation complex, was increased significantly in Rapa-2 males and Rapa females.

A decrease in the overall rate of protein synthesis by Rapa could be important in translation fidelity and protein quality. Conn and Qian [44] recently reported that activation of mTOR activity by Rheb led to an increased rate of ribosomal translation and reduced translation fidelity, while inhibition of mTOR by Rapa restored translation fidelity by reducing the rate of ribosome translation of mRNA [45]. Our observation that Rapa alters eIF2 signaling and the regulation of eIF4 & p70S6K signaling pathways are consistent with the suggestion by Kaeberlein and Kennedy [46] that reduction of global mRNA translation and increase in selective transcripts may be beneficial in aging, and Selman et al. [17] reported that null mutation in the S6k1 gene increases the lifespan of female, but not male mice. It is also interesting to note that Rapa did not extend the lifespan of *Drosophila* that express a constitutively active form of S6K1 or in *Drosophila* null in 4E-BP1 [16], indicating that down-regulation of protein synthesis may be important in the lifespan extension effects of Rapa in *Drosophila*. Therefore, the alterations we have observed in the eIF2 signaling and the regulation of eIF4 & p70S6K signaling pathways could potentially contribute to the life-extending actions of Rapa.

One interesting observation from our study was that ERK signaling was common to 10 of the 13 IPA pathways found to be shared in Rapa-2 males and Rapa females. Based on the increase in the transcripts for *c-Raf* and *Erk1/2* in Rapa-2 males and for *c-Raf, Mek1/2*, and *Erk* in the Rapa females, it would appear that ERK signaling is increased in the Rapa treated mice and that this increase might be important in the changes we observe in some of these pathways. For example, chronic activation of ERK signaling has been shown to down-regulate the NRF2 pathway [47]. Also, glucocorticoids have been shown to inhibit ERK signaling and because the glucocorticoid receptor signaling is down-regulated by Rapa, this could be a potential reason in the increase in ERK signaling [48]. ERK signaling may be increased to counteract the decrease in protein synthesis by Rapa, which is known to decrease protein synthesis, because ERK signaling has been shown to regulate ribosome biogenesis [49] and S6k1 [50].

In summary, our study provides the first insight into the potential mechanisms of Rapa's life-extending mechanisms. We identified several pathways, which would have been predicted from what is known about rapamycin's mode of action, e.g., alterations in pathways involved in protein synthesis and turnover. Our data suggest that Rapa improves protein quality by reducing protein synthesis and shifting protein degradation to autophagy. Improved protein quality has been proposed to play a major role in aging [51], [52]. On the other hand, the alterations we observed in the apparent reduction in pathways involved in protecting cells/tissues from stress (NRF2-mediated oxidative stress response), as well as reduced levels of transcripts for several antioxidant enzymes, were surprising and suggest that animals fed Rapa are more sensitive to stress, including oxidative stress. Research over the past two decades has shown that almost all manipulations that increase lifespan show increased resistance to stress, e.g., DR, dwarf mouse models, and the many long-lived mutants of *C. elegans* [53]. If future studies confirm our pathway analysis of the transcriptome and show that tissues/cells from mice fed Rapa are more sensitive, or even have no change in sensitivity to stress, Rapa would be one of the few manipulations that increase lifespan without a corresponding increase in stress resistance, suggesting that combining Rapa with other manipulations might extend lifespan further. Finally, we were surprised to find that the pathway most robustly affected by Rapa was mitochondrial function. While mitochondrial function has long been viewed as important in aging, there is currently little evidence that Rapa alters mitochondria function. Our transcriptome data indicate that a more intensive study of the effect of Rapa/mTOR on mitochondrial function is needed.

## Supporting Information

File S1 **Supporting information file containing Figures S1–S7, Tables S1–S4, supplementary methods, and detail descriptions of the tabs in File 2, 3, and 4.** Figure S1: Body weight data for mice from 5 to 21 months of age. Figure S2: Food consumption data for mice from 5 to 24 months of age. Table S1: Survival analysis of C57BL/6 mice fed Rapamycin. Table S2: Fitted Gompertz Mortality Models. Table S3: Tissue Weights at 25 months of age. Figure S3: Phosphorylated S6/Total S6 ratio shows no change among all the groups. Figure S4: Gene analysis shows that Rapa-2 males share many genes that change in Rapa Female mice. Figure S5: Validation of genes in the mitochondria dysfunction and estrogen receptor signaling pathways. Table S4: Gene enrichment analysis using DAVID bioinformatics resources. Figure S6: Gene analysis shows significant gene changes in 6-months Rapa female mice. Figure S7: Fibroblasts pretreated with Rapa are more sensitive to oxidative stressors.
(XLSX)

File S2 **File containing data for genes and pathways found to be significantly changed by Rapa.**
(XLSX)

File S3 **File containing data for genes significantly changed in pathways found to be shared by Rapa-2 male and Rapa female.**
(XLSX)

File S4 **File containing data for DAVID gene enrichment analysis ranked by enrichment score using genes significantly changed in chronic Rapa-2 male, chronic Rapa female, and 6-months Rapa females.**
(XLSX)
