# Correction: Mice Fed Rapamycin Have an Increase in Lifespan Associated with Major Changes in the Liver Transcriptome

**DOI:** 10.1371/journal.pone.0092346

**Published:** 2014-04-01

**Authors:** The PLOS ONE Staff

File S1 is a duplicate of File S2. The correct File S1 can be found here.

## Supporting Information

File S1 **Supporting information file containing Figures S1–S7, Tables S1–S4, supplementary methods, and detail descriptions of the tabs in File 2, 3, and 4.** Figure S1: Body weight data for mice from 5 to 21 months of age. Figure S2: Food consumption data for mice from 5 to 24 months of age. Table S1: Survival analysis of C57BL/6 mice fed Rapamycin. Table S2: Fitted Gompertz Mortality Models. Table S3: Tissue Weights at 25 months of age. Figure S3: Phosphorylated S6/Total S6 ratio shows no change among all the groups. Figure S4: Gene analysis shows that Rapa-2 males share many genes that change in Rapa Female mice. Figure S5: Validation of genes in the mitochondria dysfunction and estrogen receptor signaling pathways. Table S4: Gene enrichment analysis using DAVID bioinformatics resources. Figure S6: Gene analysis shows significant gene changes in 6-months Rapa female mice. Figure S7: Fibroblasts pretreated with Rapa are more sensitive to oxidative stressors.
doi:10.1371/journal.pone.0083988.s001
(PDF)
