# Supplementary material for: Correction: Mice Fed Rapamycin Have an Increase in Lifespan Associated with Major Changes in the Liver Transcriptome
Source: PLoS One. 2014 Apr 1;9(4):e92346. doi: 10.1371/journal.pone.0092346 (PMC3972154; doi:10.1371/journal.pone.0092346)
Supplement: File S1 — Supporting information file containing Figures S1–S7, Tables S1–S4, supplementary methods, and detail descriptions of the tabs in File 2, 3, and 4. Figure S1: Body weight data for mice from 5 to 21 months of age. Figure S2: Food consumption data for mice from 5 to 24 months of age. Table S1: Survival analysis of C57BL/6 mice fed Rapamycin. Table S2: Fitted Gompertz Mortality Models. Table S3: Tissue Weights at 25 months of age. Figure S3: Phosphorylated S6/Total S6 ratio shows no change among all the groups. Figure S4: Gene analysis shows that Rapa-2 males share many genes that change in Rapa Female mice. Figure S5: Validation of genes in the mitochondria dysfunction and estrogen receptor signaling pathways. Table S4: Gene enrichment analysis using DAVID bioinformatics resources. Figure S6: Gene analysis shows significant gene changes in 6-months Rapa female mice. Figure S7: Fibroblasts pretreated with Rapa are more sensitive to oxidative stressors. doi:10.1371/journal.pone.0083988.s001 (PDF) [file pone.0092346.s001.pdf]

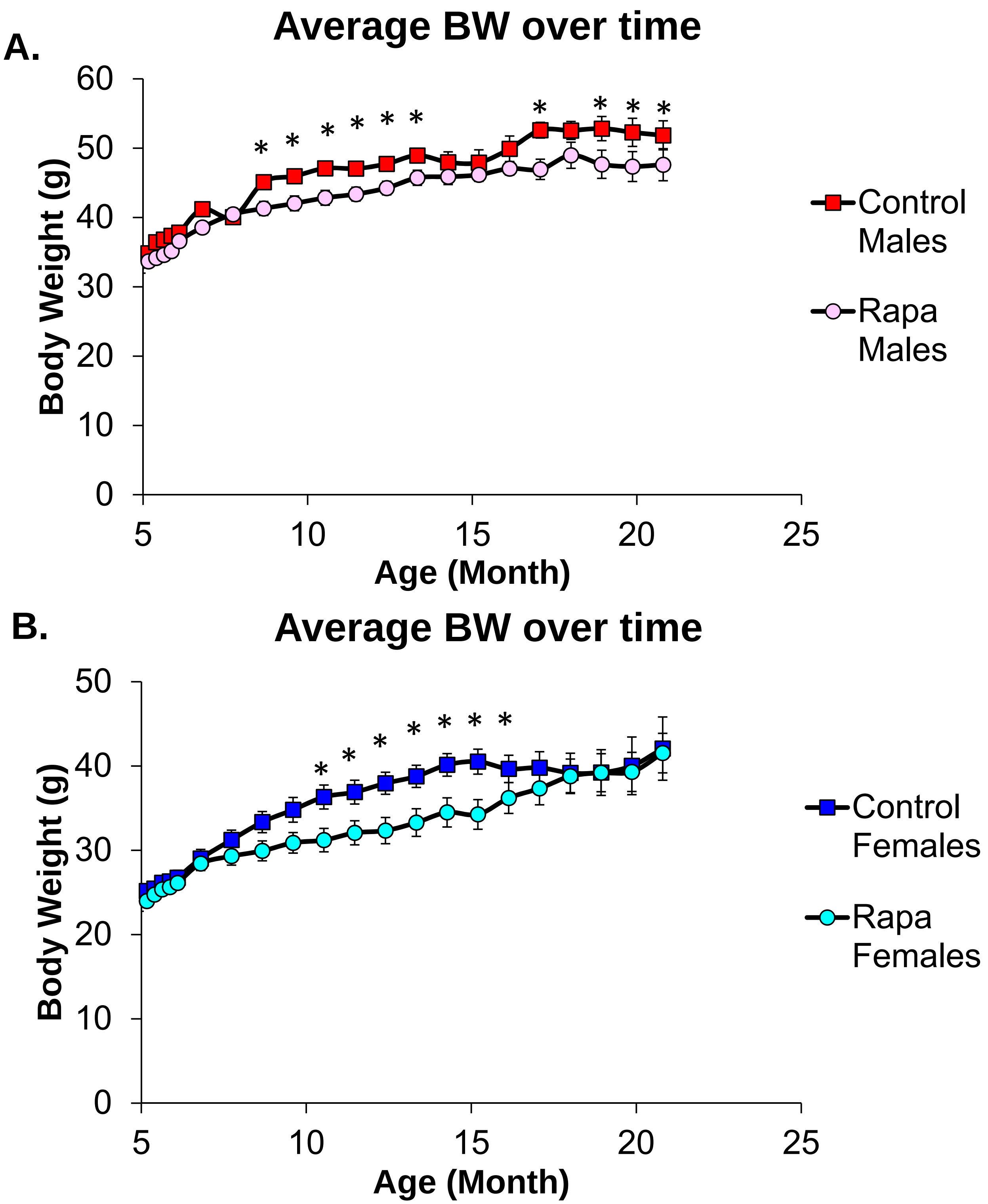

Figure S1: Body weight data for mice from 5 to 21 months of age.
Body weight data for control and rapamycin (Rapa) male **(A)** and control and Rapa female **(B)** mice are shown from 5 to 21 months of age for 15 mice in each group. The data are the mean ± standard error of the mean (SEM) statistically analyzed by Two-way ANOVA with repeated measure followed by Tukey's pairwise comparisons. The data that were statistically significant with $p<0.05$ are indicated by *.

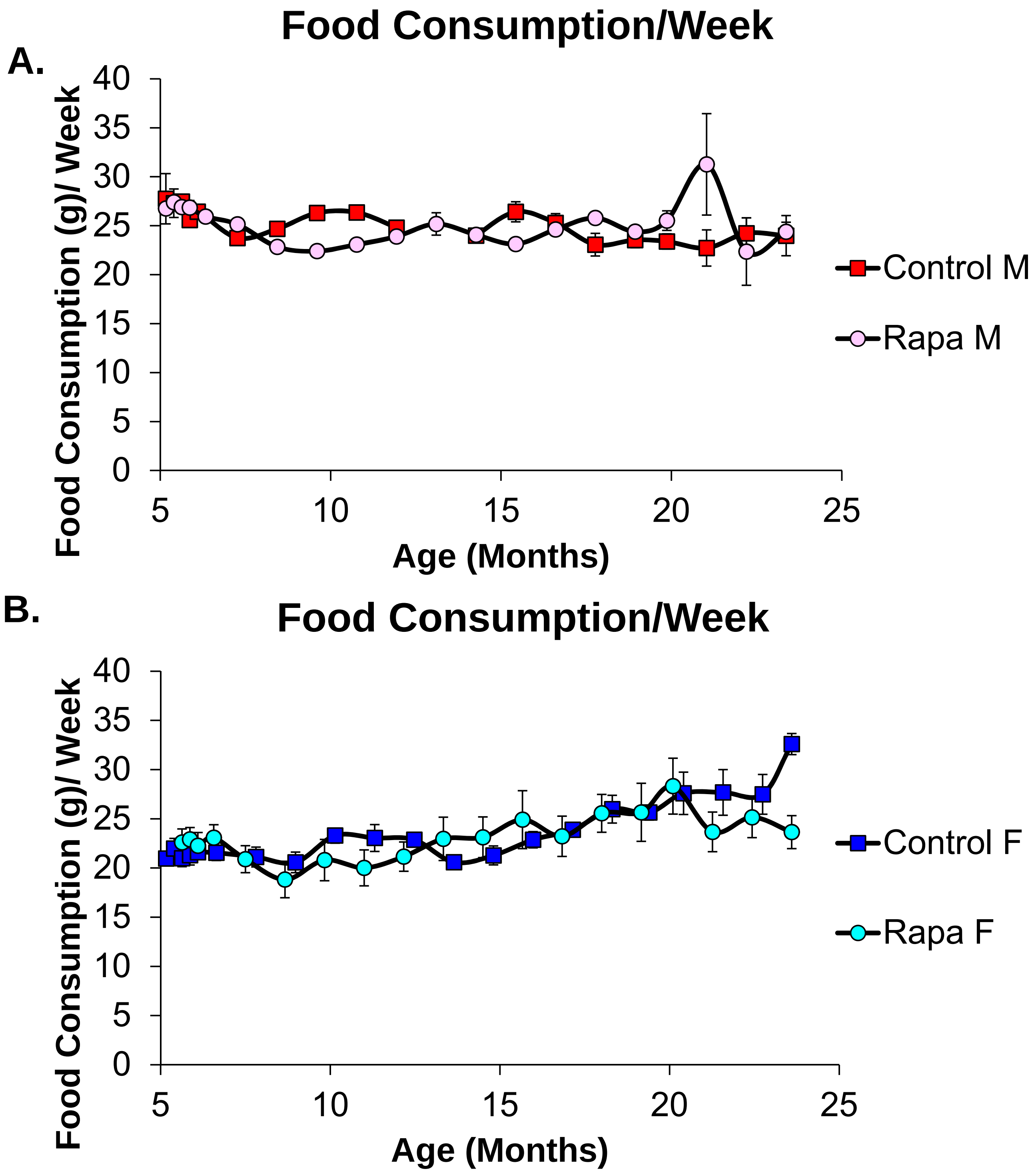

Figure S2: Food consumption data for mice from 5 to 24 months of age. Food consumption data is presented as average consumption per week per mouse for control and Rapa male **(A)** and control and Rapa female **(B)** mice from 5 to 24 months of age. Food consumption was measured per week minus the food not consumed at the bottom of the cage and calculated per mice in the cage. Food consumption was measured for 3 cages per group with 5 mice in each cage. The data are the mean ± SEM and were statistically analyzed by Two-way ANOVA with repeated measure follow by Tukey's pairwise comparisons. We did not observe any statistical difference between Rapa and control for both males and females.

		Mean + SEM	80% Survival	50% Survival	10% Survival	Max
Males	Control	806 ± 24 (756, 856)	726 (661, 776)	804 (776, 863)	991 (937, 1059)	1097
	Rapa	864 ± 30 (803, 925)	680 (549, 843)	917 (843, 961)	1074 (1046, 1159)	1178
	p-value	p=0.149	p=0.871	p=0.014	p=0.037	
	adjusted p-value	p=0.268	p=0.875	p=0.056	p=0.111	
Females	Control	826 ± 21 (783, 869)	708 (623, 793)	865 (793, 933)	965 (953, 1031)	1048
	Rapa	945 ± 22 (899, 991)	834 (820, 917)	928 (917, 971)	1153 (1075, 1177)	1231
	p-value	p<0.001	p=0.003	p=0.010	p=0.004	
	adjusted p-value	p<0.001	p=0.009	p=0.010	p=0.009	

Table S1: Survival analysis of C57BL/6 mice fed Rapamycin.
The mean, 80%, 50%, 10% (when 90% of the mice in the group are dead), and maximum (when last mouse in the group dies) survival of the mice in each group are show in days for 45 mice per group except for control males, which had 40 mice. The SEM for the mean and 95% confidence intervals for the mean, 80%, 50%, and 10% survival are given in the parenthesis. The effect of rapamycin on each parameter was statistically compared using a bootstrapping method and the p-values and adjusted p-values are given.

	Gompertz *a* (Initial Mortality Rate)	Chi-Square (*a*)	Gompertz *b* (Rate of increase in hazard for mortality)	Chi-Square (*b*)	Chi-Square (whole model)
Control Female	0.00021 (0.00004, 0.00106)	p=0.689	0.2803 (0.2198, 0.3575)	p=0.159	p<0.001
Rapa Female	0.00032 (0.00008, 0.00129)		0.2215 (0.1765, 0.2780)		
Control Male	0.00066 (0.00017, 0.00255)	p=0.655	0.2320 (0.1815, 0.2965)	p=0.252	p=0.004
Rapa Male	0.00099 (0.00029, 0.00334)		0.1894 (0.1474, 0.2434)		
Control (Both sex)	0.00039 (0.00014, 0.00111)	p=0.534	0.2537 (0.2135, 0.3014)	p=0.064	p<0.001
Rapa (Both sex)	0.0006 (0.00024, 0.00150)		0.2029 (0.1715, 0.2340)		

Table S2: Fitted Gompertz Mortality Models

Ln plots of fitted Gompertz mortality models where the hazard (instantaneous mortality rate) at age x, $u_x = ae^{bx}$, where x begins at 4 months and continues until the end of life. The parameters for the Gompertz model are shown with Gompertz parameter *a* as the initial mortality rate at 4 months and, Gompertz *b* as the rate of increasing mortality. In the parenthesis are the 95% confidence interval for parameters Gompertz *a* and *b*. Statistical analysis was done using maximum-likelihood estimation and generating chi-square tests for comparison of constrained and freely varying models. The p-values indicating the level of confidence in equating model parameters are given. We found that the whole mortality model differed between Rapa and Control in both females (p<0.0001) and males (p=0.0039). Pooling of the sexes allowed for identifying the parameter(s) that differ between Rapa and control-fed animals, and indicates that the rate of aging (*b*), but not the initial mortality rate (*a*), differed between Rapa and control mice.

Tissue		Control Male	Rapa Male	Control Female	Rapa Female
Brain		0.476±0.004	0.478±0.006	0.480±0.005	0.480±0.004
Heart		0.157±0.007	0.168±0.018	0.170±0.007	0.145±0.006
Liver		2.349±0.171	2.360±0.195	1.877±0.185	1.787±0.091$^{\&}$
Kidney		0.504±0.013	0.496±0.015	0.541±0.016	0.476±0.019*
Spleen		0.135±0.0260†	0.113±0.009$^{\&}$	0.283±0.039	0.134±0.012*
Fat Depot	Epididymal Fat	1.965±0.398	1.110±0.144†	2.272±0.558	3.060±0.454†
	Perirenal Fat	0.819±0.190	0.440±0.084	0.703±0.172	0.971±0.148
	Inguinal Fat	1.063±0.201	0.719±0.133	0.955±0.228	1.340±0.15
	Subscapular Fat	0.470±0.092	0.339±0.045	0.478±0.102	0.623±0.069
	Mesenteric Fat	0.530±0.133	0.403±0.075	0.366±0.094	0.692±0.108
	Brown Fat	0.153±0.018	0.161±0.019	0.166±0.019	0.180±0.012
Skeletal Muscle	Gastrocnemius	0.301±0.010†	0.287±0.0110$^{\&}$	0.221±0.012	0.251±0.010$^{\&}$
	Quadriceps	0.385±0.014†	0.386±0.013$^{\&}$	0.288±0.017	0.291±0.014$^{\dagger,\&}$
	Soleus	0.018±0.001†	0.017±0.001$^{\&}$	0.013±0.001	0.016±0.001
	Tibialis Anterior	0.186±0.005†	0.180±0.006$^{\dagger,\&}$	0.131±0.005	0.139±0.005†
Spinal cord		0.108±0.003	0.116±0.006	0.103±0.006	0.147±0.039

*$p<0.05$ to same sex Control
$^{\dagger}p<0.05$ to same group in opposite sex
$^{\&}p<0.05$ to opposite sex control

Table S3: Tissue Weights at 25 months of age
The mean ± SEM weights of various tissues from 25-month-old male and female control and Rapa mice are shown in the table. The data were analyzed using one-way ANOVA with Tukey's pairwise comparisons and those values that are significantly different at $p<0.05$ are indicated by * compared to same sex control, † compared to the same group in opposite sex, and $^{\&}$ compared to the opposite sex control.

Male:

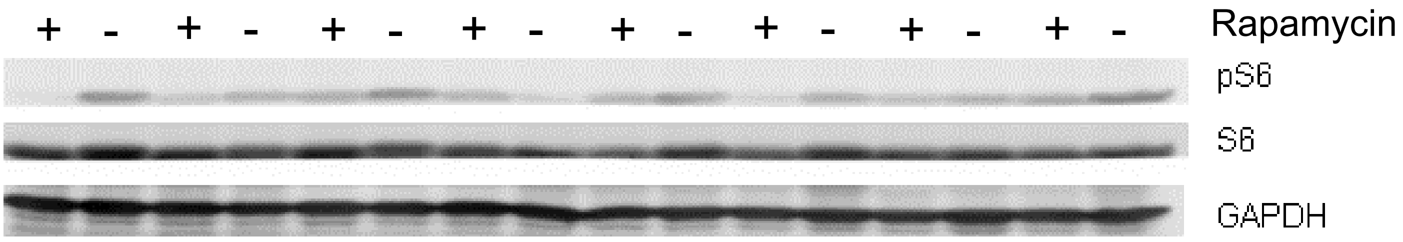

Female:

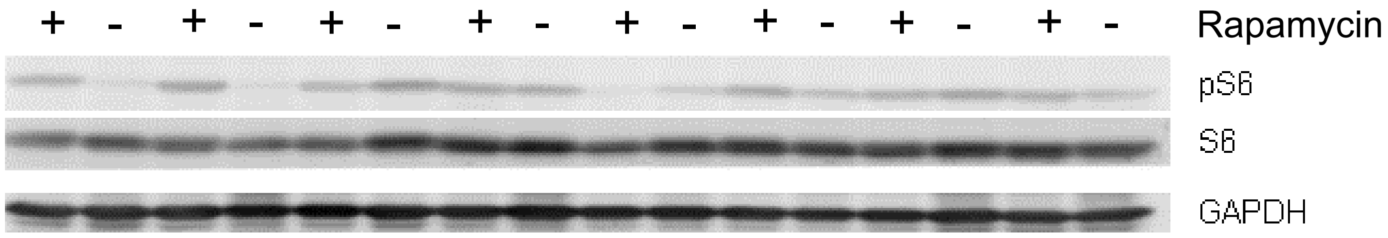

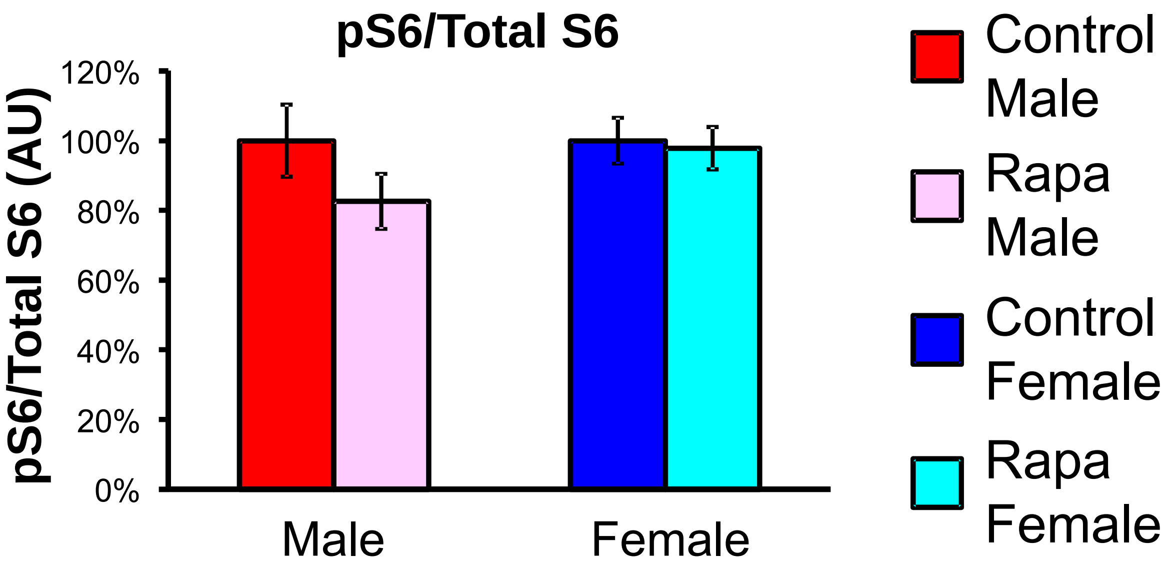

Figure S3: Phosphorylated S6/Total S6 ratio shows no change among all the groups. mTOR signaling measured by phosphorylated S6/Total S6 is shown for male and female control and Rapa mice**.** The data shown are mean ± SEM for 12 mice each in control and Rapa males and 16 mice each in control and Rapa females.

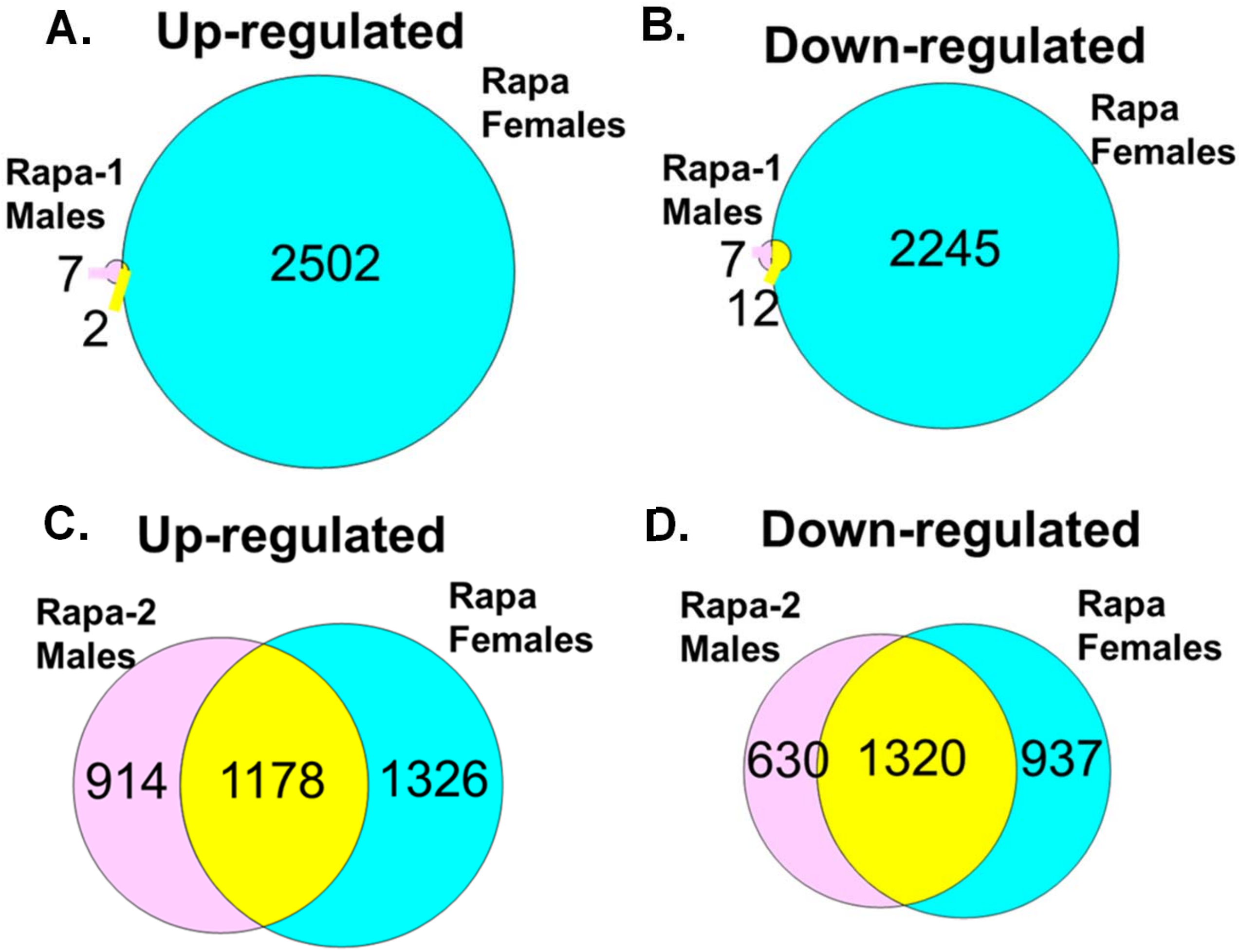

Figure S4: Gene analysis shows that Rapa-2 males share many genes that change in Rapa Female mice.
We identified the transcripts that were significantly changed by Rapa using a false discovery rate of adjusted p-value <0.05 and $>15\%$ change relative to respective controls for each sex. As described in Figure 2, the male mice fed Rapa appeared in analysis to segregate as 2 groups, which will be shown as Rapa-1 and Rapa-2. The venn diagrams show the number of transcripts that were significantly up-regulated **(A)** or down-regulated **(B)**, in Rapa-1 male and Rapa female mice, and the transcripts that were significantly up-regulated **(C)** and down-regulated **(D)** in Rapa-2 male and Rapa female mice. The Rapa male mice are represented in pink, Rapa female mice in cyan, and the yellow indicates sharing of Rapa male and female mice.

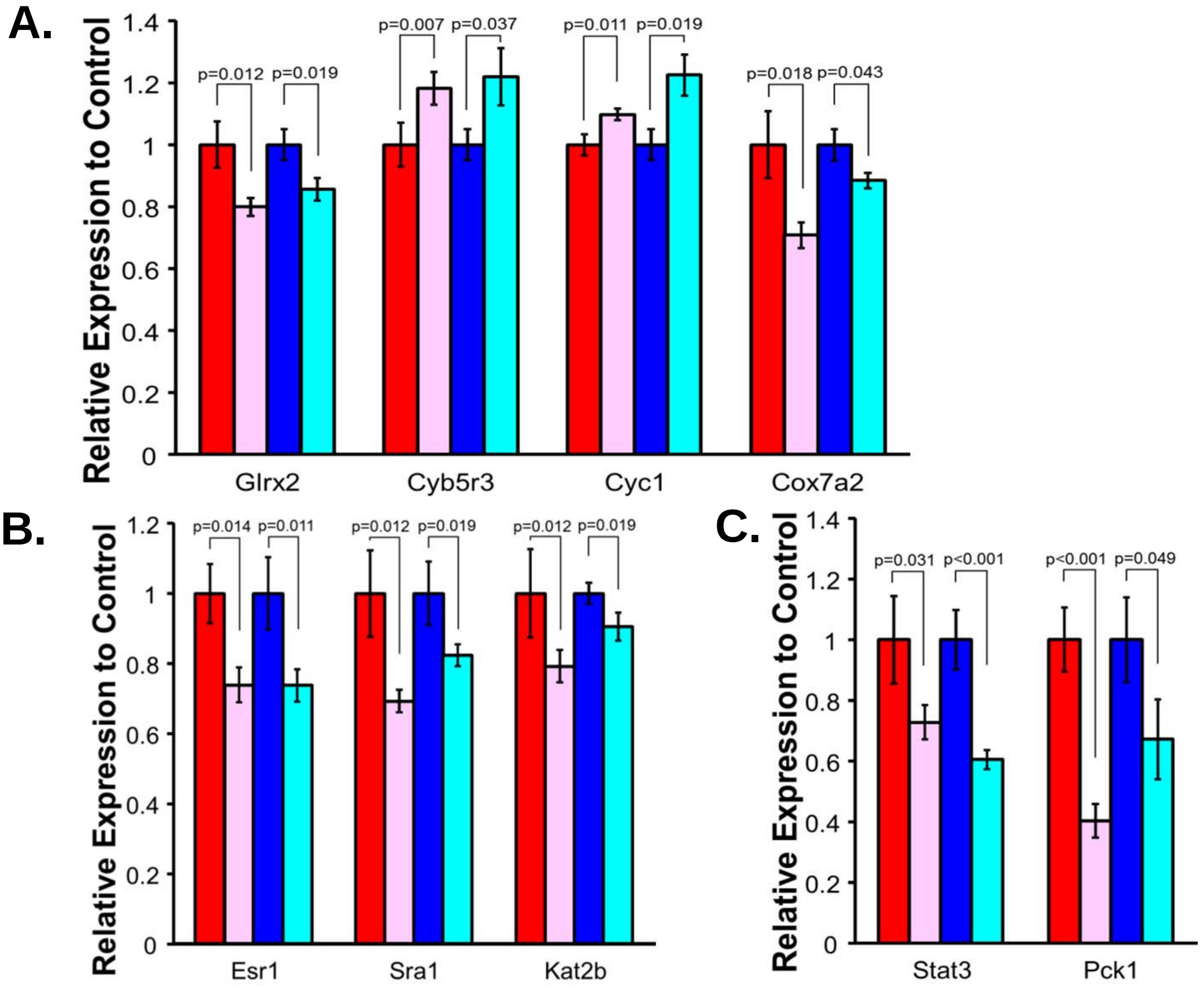

D.

Gene	5' primer	3' primer
Hprt1	GAGAGCGTTGGGCTTACCTC	CTAATCACGACGCTGGGACT
Esr1	ACATGCCTATTGCTGGGTGT	TGACCCATTAGCTACTGTCCAA
Sra1	GTCAGTCAGTGGATGGTGGG	GGCTGTTGGAAGCCTGGTAT
Kat2b	GCTTGGCCTTTCATGGAACC	TGTTCCTGAGGCGTTCACTC
Stat3	TGGCTCTGCACTTTCAACCT	GTGTCAGTGTGTTTTGCCCT
Pck1	TGGGGAAAAATCTTGGGCAAA	AGAAAGGTAAGGAAGGGCGG
Glrx2	TGCTGGAATATGGCAACCAGT	CGCTGCGCCTCCAATAAATC
Cyb5r3	GCCTGTGCCCCCTAAACACT	CGGGGGCAGTGTGTATAGTTT
Cyc1	CTGGGGTGTCATTGCGAGAA	TTGTGACATGGTAGCTGGGG
Cox7a2	CGCTCGATGGACCAGGAATC	TGCCAACTGCTGACACAAAA

Figure S5: Validation of genes in the mitochondria dysfunction and estrogen receptor signaling pathways.

Using qRT-PCR, we measured the levels selected transcripts that were observed to change in the mitochondria dysfunction **(A)** and estrogen receptor signaling **(B, C)** pathways. The data shown are the means relative to respective sex controls ± SEM. The data were analyzed using student t-test, and the p-values are shown. For the colors, Control male is in red, Rapa-2 male in pink, control female in blue and Rapa female in cyan. A list of the primers used in the qRT-PCR validation is listed **(D)**.

A.

Chronic Rapa-2 Male up-regulated genes	
**Enrichment Score**	**Summarized GO Terms**
23.9	Lumen
10.4	Protein Degradation
10.3	mRNA processing
9.5	protein trafficking
8.7	mitochondria

B.

Chronic Rapa-2 Male down-regulated genes	
**Enrichment Score**	**Summarized GO Terms**
17.6	Lumen
17.5	mitochondria
10.3	mitochondrial membrane
9.1	protein degradation
8.8	mitochondria

C.

Chronic Rapa female up-regulated genes	
**Enrichment Score**	**Summarized GO Terms**
41.6	mitochondria
25.6	mitochondrial membrane
13.7	Lumen
11.1	protein trafficking
8.1	mRNA processing

D.

Chronic Rapa female down-regulated genes	
**Enrichment Score**	**Summarized GO Terms**
12.2	Lumen
8.8	mitochondria
7.8	Protein Degradation
4.4	Ribosomes
4.3	RNA binding proteins

E.

6-months Rapa female up-regulated genes	
**Enrichment Score**	**Summarized GO Terms**
12.5	Protein Degradation
7.7	protein trafficking
7.4	Lumen
7.2	ATP/nucleotide binding
4.7	Ubiquitin

F.

6-months Rapa female down-regulated genes	
**Enrichment Score**	**Summarized GO Terms**
14.1	mitochondria
7.5	mitochondrial membrane
7.2	mitochondria
4.6	Proteasome
4.3	Unfolded protein response

Table S4: Gene enrichment analysis using DAVID bioinformatics resources.
Liver transcriptome analysis using genes significantly changed were analyzed using DAVID bioinformatics resources. The top 5 enrichment scores for the summarized gene ontology terms are listed for chronic Rapa-2 males using up-regulated genes **(A)**, down-regulated genes **(B)**, chronic Rapa females using up-regulated genes **(C)**, down-regulated genes **(D)**, 6-months Rapa females using up-regulated **(E)**, and down-regulated genes **(F)** are shown ranked by enrichment score. A full list of the gene enrichment as well as the expanded gene ontology list for each enrichment cluster is shown in File S4 Tab1, Tab2, Tab3, Tab4, Tab5, and Tab6.

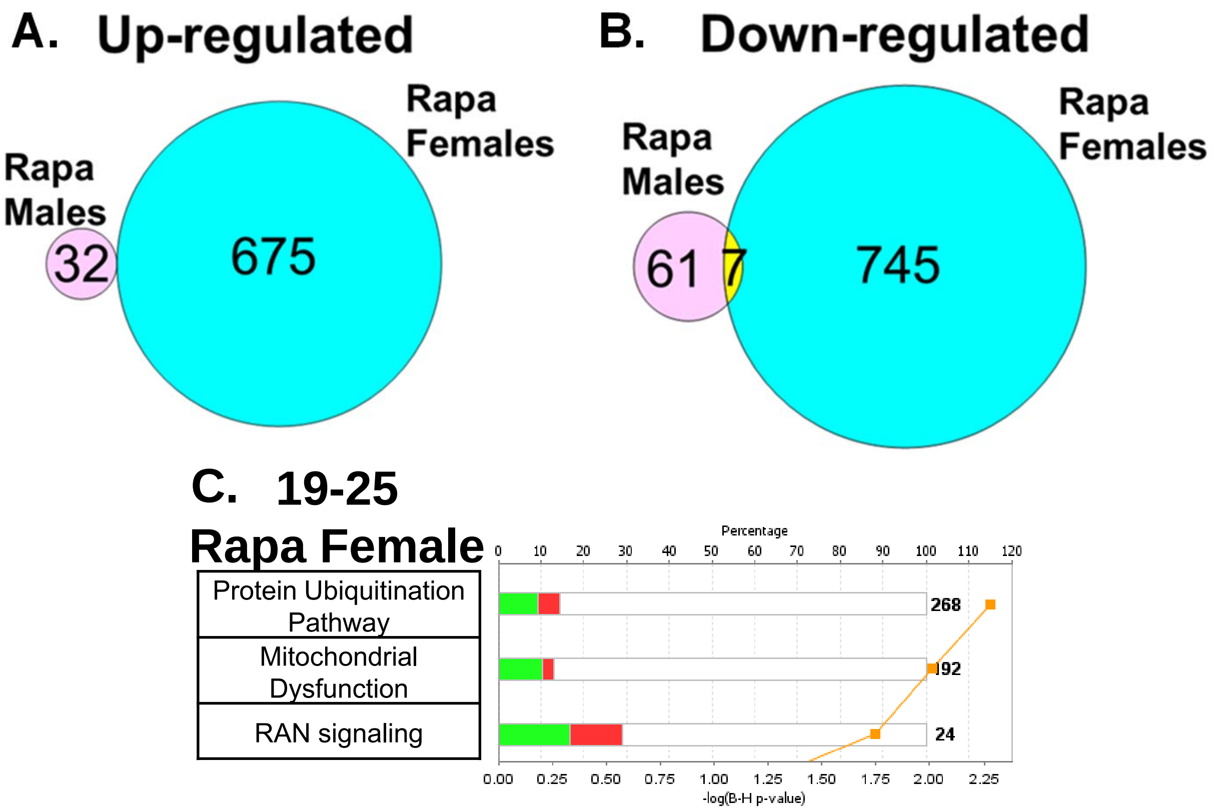

Figure S6: Gene analysis shows significant gene changes in 6-months Rapa female mice.

We identified the transcripts that were significantly changed by 6 months of Rapa treatment using a false discovery rate of adjusted p-value <0.05 and >15% change relative to respective controls for each sex. The venn diagrams show the number of transcripts that were significantly up-regulated **(A)** or down-regulated **(B)**, in 6-months Rapa male and Rapa female mice. The Rapa male mice are represented in pink, Rapa female mice in cyan, and the yellow indicates sharing of Rapa male and female mice. Pathway analysis was conducted using ingenuity pathway analysis for genes that were significantly changed in 25-month-old females fed Rapa for 6 months relative to control females with adjusted p<0.05 and >15% change **(C)**. The pathway analysis used Fisher's exact test to place genes into pathways and we also used a FDR cutoff of B-H p<0.05 to show the pathways that were highly significant. All the pathways (3) with a B-H p<0.05 are shown. Two of the three pathways, protein ubiquitination and mitochondrial dysfunction, are also changed in chronic Rapa female. For the graphical portion, the yellow line indicates the –log(B-H p-value). Red indicates up-regulated and green for down-regulated genes in our transcriptome analysis found to be significantly changed in Rapa compared to control, and white indicates the percentage of genes not significantly changed in the pathway relative to the total number of genes in that pathway (bolded number). A list of the pathways that significantly changed with 6-months Rapa feeding is shown in in File S2, tab5 for Rapa female and a list of the transcripts that change in protein ubiquitination and mitochondrial dysfunction is shown in File S3 tab14 and tab15 for 6-months Rapa female compared to chronic Rapa female.

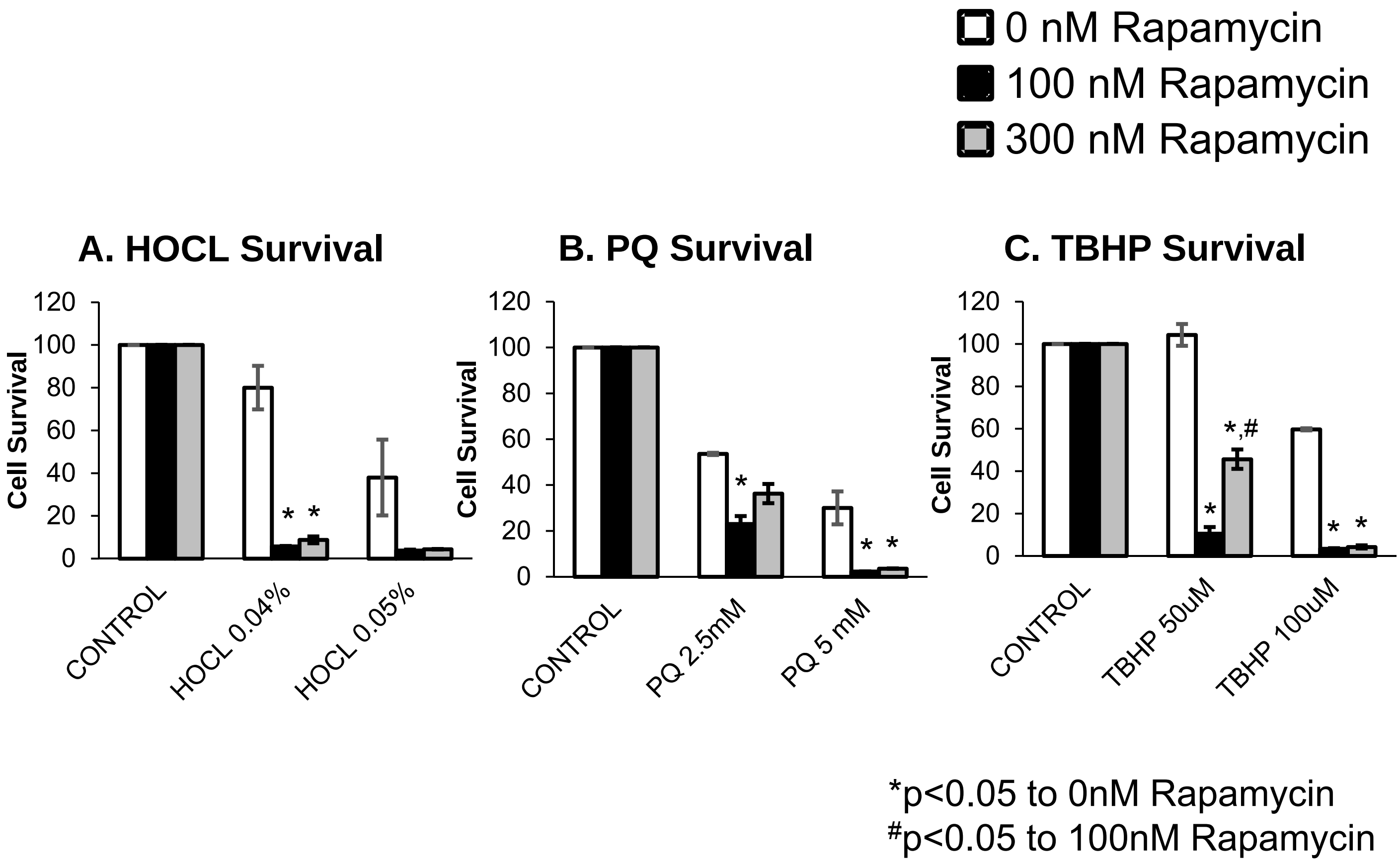

Figure S7: Fibroblasts pretreated with Rapa are more sensitive to oxidative stressors. Mouse primary dermal fibroblasts from 3-6 month old C57BL/6 mice at passage 2 were pretreated with 0 (white bars), 100 (black bars) or 300 (grey bars) nM of Rapa for 24 hours. Cells were then washed and the media was then replaced. Cells were treated with hypochlorous acid at 0.04% and 0.05% **(A)**, paraquat at 2.5mM and 5 mM **(B)**, and tert-Butyl hydroperoxide at 50μM and 100μM **(C)** for 6 hours after which cells were washed and media was replaced. After 18 hours, cell viability was determined using a microplate reader. The data are the mean ± SEM are shown for 2 independent of replicates. The asterisk indicates $p<0.05$ to 0 nM Rapa and the number sign indicates $p<0.05$ to 100 nM. Addition of Rapa significantly decreases cell survival with all the stressors.

File S2: File (File S2.xlsx) containing data for genes and pathways found to be significantly changed by Rapa.

Tab1 ("1. Genes-Chronic Rapa"): A list of all genes found to be significantly different in chronic Rapa male or female (using criteria of adjusted p<0.05) from respective sex controls. The list is sorted in the order of the gene hierarchical cluster of the heatmap in Figure 3. The group column indicates where the genes are found in the Venn diagram (Figure S4A-S4D). The log change is colored with red for significantly up-regulated genes, green for significantly down-regulated genes, and white for non-significant genes.

Tab2 ("2. IPA Rapa-2 Male|Control Male"): IPA pathways for genes significantly altered in chronic Rapa-2 male/control male with a B-H p<0.05, ranked from lowest to the highest B-H p-value.

Tab3 ("3. IPA Rapa Female|Control F"): IPA pathways for genes significantly altered in Rapa female/control female with a B-H p<0.05, ranked from lowest to the highest B-H p-value.

Tab4 ("4. Genes-6-months Rapa"): A list of all genes found to be significantly different in 6-months Rapa male or female (using criteria of adjusted p<0.05) from respective sex controls. The list is sorted in the order of the gene hierarchical cluster of the heatmap in Figure 6B. The group column indicates where the genes are found in the Venn diagram (Figure S6A and S6B). The log change is colored with red for significantly up-regulated genes, green for significantly down-regulated genes, and white for non-significant genes.

Tab5 ("5. IPA 6-months Rapa F|Ctrl F"): IPA pathways for genes significantly altered in 6-months Rapa female/control female with a B-H p<0.05, ranked from lowest to the highest B-H p-value.

Tab6 ("6. Genes- 6-mon vs. Chronic Rapa"): A list of all genes found to be significantly different in 6-months Rapa female or chronic Rapa female (using criteria of adjusted p<0.05) from respective controls and matched from both microarray sets. The list is sorted in the order of the gene hierarchical cluster of the heatmap in Figure 7A. The group column indicates which group the gene is found significant in. The log change is colored with red for significantly up-regulated genes, green for significantly down-regulated genes, and white for non-significant genes.

File S3: File (File S3.xlsx) containing data for genes significantly changed in pathways found to be shared by Rapa-2 male and Rapa female.

Tab1 ("1. Estrogen Receptor S. 04-25"): A list of all genes for estrogen receptor signaling pathway found to be significantly changed in chronic Rapa-2 male or Rapa female compare to respective controls. Red indicates up-regulated genes, green indicates down-regulated genes, and white indicates no change.

Tab2 ("2. VEGF Signaling 04-25"): A list of all genes for VEGF signaling pathway found to be significantly changed in chronic Rapa-2 male or Rapa female compare to respective controls. Red indicates up-regulated genes, green indicates down-regulated genes, and white indicates no change.

Tab3 ("3. Chronic Myeloid L. 04-25"): A list of all genes for Chronic Myeloid Leukemia signaling pathway found to be significantly changed in chronic Rapa-2 male or Rapa female compare to respective controls. Red indicates up-regulated genes, green indicates down-regulated genes, and white indicates no change.

Tab4 (“4. Telomerase Signaling 04-25”): A list of all genes for Telomerase signaling pathway found to be significantly changed in chronic Rapa-2 male or Rapa female compare to respective controls. Red indicates up-regulated genes, green indicates down-regulated genes, and white indicates no change.

Tab5 (“5. Integrin Signaling 04-25”): A list of all genes for Integrin signaling pathway found to be significantly changed in chronic Rapa-2 male or Rapa female compare to respective controls. Red indicates up-regulated genes, green indicates down-regulated genes, and white indicates no change.

Tab6 (“Mito Function- 04-25 M2 vs. F”): A list of all genes for mitochondria dysfunction pathway found to be significantly changed in chronic Rapa-2 male or Rapa female compare to respective controls. Red indicates up-regulated genes, green indicates down-regulated genes, and white indicates no change.

Tab7 (“7. NRF2 Signaling 04-25”): A list of all genes for NRF2-mediated oxidative stress response pathway found to be significantly changed in chronic Rapa-2 male or Rapa female compare to respective controls. Red indicates up-regulated genes, green indicates down-regulated genes, and white indicates no change.

Tab8 (“8. Protein Ubiquitination 04-25”): A list of all genes for protein ubiquitination pathway found to be significantly changed in chronic Rapa-2 male or Rapa female compare to respective controls. Red indicates up-regulated genes, green indicates down-regulated genes, and white indicates no change.

Tab9 (“9. IGF-1 Signaling 04-25”): A list of all genes for IGF-1 signaling pathway found to be significantly changed in chronic Rapa-2 male or Rapa female compare to respective controls. Red indicates up-regulated genes, green indicates down-regulated genes, and white indicates no change.

Tab10 (“10. Glucocorticoid R. 04-25”): A list of all genes for glucocorticoid receptor signaling pathway found to be significantly changed in chronic Rapa-2 male or Rapa female compare to respective controls. Red indicates up-regulated genes, green indicates down-regulated genes, and white indicates no change.

Tab11 (“11. eIF2 Signaling 04-25”): A list of all genes for eIF2 signaling pathway found to be significantly changed in chronic Rapa-2 male or Rapa female compare to respective controls. Red indicates up-regulated genes, green indicates down-regulated genes, and white indicates no change.

Tab12 (“12. eIF4 Signaling 04-25”): A list of all genes for regulation of eIF4 & p70S6K signaling pathway found to be significantly changed in chronic Rapa-2 male or Rapa female compare to respective controls. Red indicates up-regulated genes, green indicates down-regulated genes, and white indicates no change.

Tab13 (“13. Tryptophan D. 04-25”): A list of all genes for tryptophan degradation pathway found to be significantly changed in chronic Rapa-2 male or Rapa female compare to respective controls. Red indicates up-regulated genes, green indicates down-regulated genes, and white indicates no change.

Tab14 (“14. Mito Function- RF6 vs RFL”): A list of all genes for mitochondria dysfunction pathway found to be significantly changed in 6-months Rapa female or chronic Rapa female compare to respective controls. Red indicates up-regulated genes, green indicates down-regulated genes, and white indicates no change.

Tab15 (“15. Protein Ubiq. RF6 vs RFL”): A list of all genes for protein ubiquitination pathway found to be significantly changed in 6-months Rapa female or chronic Rapa female compare to respective controls. Red indicates up-regulated genes, green indicates down-regulated genes, and white indicates no change.

File S4: File (File S4.xlsx) containing data for DAVID gene enrichment analysis ranked by enrichment score using genes significantly changed in chronic Rapa-2 male, chronic Rapa female, and 6-months Rapa females.

Tab1 (“1. Chronic Rapa-2 male up”): DAVID functional annotation cluster analyzed using genes significantly up-regulated in chronic Rapa-2 males.

Tab2 (“2. Chronic Rapa-2 male down”): DAVID functional annotation cluster analyzed using genes significantly down-regulated in chronic Rapa-2 males.

Tab3 (“3. Chronic Rapa female up”): DAVID functional annotation cluster analyzed using genes significantly up-regulated in chronic Rapa females.

Tab4 (“4. Chronic Rapa female down”): DAVID functional annotation cluster analyzed using genes significantly down-regulated in chronic Rapa females.

Tab5 (“5. 6-Months Rapa female up”): DAVID functional annotation cluster analyzed using genes significantly up-regulated in 6-Months Rapa females.

Tab6 (“6. 6-Months Rapa female down”): DAVID functional annotation cluster analyzed using genes significantly down-regulated in 6-Months Rapa females.

Supplementary Methods:

mTOR Signaling

A Dounce homogenizer was used to homogenize frozen liver tissue in RIPA buffer (1× PBS, 1% Nonidet P-40, 0.5% sodium deoxycholate, 0.1% SDS) supplemented with protease and phosphatase inhibitors (Roche, Indiana). The homogenate was then centrifuged at 4 °C, 12,000 x g for 10 min and the supernatant was collected. Samples were separate by SDS–PAGE and transfer to PDVF membrane. The following antibodies were used against the targeted proteins of interest for western blot analysis: actin, S6 and phospho-S6 (Ser235/236) (Cell Signaling, Danvers, MA.). mTOR signaling was assessed by the ratio of phosopho-S6 levels to total S6 levels.

Quantitative Real-Time PCR

One µg of RNA, using the total RNA extracted from frozen liver tissues described above, was used to make cDNA using the Applied Biosystems high-capacity cDNA kit following manufacturer's instructions (Life Technologies, Grand Island, NY). Primers were designed using the NCBI primer blast and were obtained from Sigma-Aldrich (St. Louis, MO). The sequences of the primers used are listed in Figure S4D. For quantitative Real Time-PCR (qRT-PCR), three dilution of cDNA (200 ng/µl, 20 ng/µl, and 2 ng/µl) in triplicate was used in the analysis of each gene using the ABI Sybrgreen Select master mix (Applied Biosystem, Inc., Foster City, CA) with 1 µl of cDNA at the indicated dilutions in a 10 µl reaction following manufacturer's protocol in ABI 7900HT qRT-PCR system (Applied Biosystem, Inc., Foster City, CA). Quantification of the qRT-PCR results was accomplished using the ΔΔCT method and gene products were assayed using agarose gels and dissociation curves. The qRT-PCR data were statistically analyzed using student t-test.

Cell Survival

Primary dermal fibroblast lines were derived from young adult (3-6 mo) C57BL/6 mice and sub-cultured in DMEM + 10% FBS + antibiotics (complete media) in a humidified incubator at 37° C, 5% CO2, and ambient oxygen. Cells that had reached confluence at passage 2 (P2) were split by trypsin digest and seeded in replicate in 96 well flat-bottom tissue culture plates at a density of 30,000 cells/well in 100 µL media. After 24 hours, media was replaced complete media containing 0, 100, or 300 nM rapamycin (LC Laboratories, Woburn MA) dissolved in DMSO. Cells were maintained an additional 24 hours under these conditions. Following rapamycin treatment, cells were washed 3X with 1X DPBS and 100 µL complete media and were treated with doses of paraquat (PQ), tert-butyl hydrogen peroxide (TBHP) or sodium hypochlorite (HOCL) in complete media for 6 hours; all stressors were purchased from Sigma (St. Louis, MO). Cells were then washed 3X with 1X DPBS and 100 µL complete media was added to all cells for 18 hours. After this time period, WST-1 (Roche, Indianapolis IN), was added to each well and plates were read on a microplate reader following manufacturer's instructions. WST-1 signal was then normalized to values obtained from untreated cells.
